# Structure and Functions of HMGB3 Protein

**DOI:** 10.3390/ijms25147656

**Published:** 2024-07-12

**Authors:** Elena Chikhirzhina, Anna Tsimokha, Alexey N. Tomilin, Alexander Polyanichko

**Affiliations:** Institute of Cytology of the Russian Academy of Sciences, Tikhoretsky Av. 4, 194064 St. Petersburg, Russia; atsimokha@incras.ru (A.T.); a.tomilin@incras.ru (A.N.T.); a.polyanichko@incras.ru (A.P.)

**Keywords:** HMGB3, protein/DNA interaction, protein structure and function

## Abstract

HMGB3 protein belongs to the group of HMGB proteins from the superfamily of nuclear proteins with high electrophoretic mobility. HMGB proteins play an active part in almost all cellular processes associated with DNA—repair, replication, recombination, and transcription—and, additionally, can act as cytokines during infectious processes, inflammatory responses, and injuries. Although the structure and functions of HMGB1 and HMGB2 proteins have been intensively studied for decades, very little attention has been paid to HMGB3 until recently. In this review, we summarize the currently available data on the molecular structure, post-translational modifications, and biological functions of HMGB3, as well as the possible role of the ubiquitin–proteasome system-dependent HMGB3 degradation in tumor development.

## 1. Introduction

HMGB3 protein belongs to a large family of non-histone chromatin proteins characterized by high electrophoretic mobility (**h**igh-**m**obility **g**roup or HMG). The protein was first discovered in chicken tissues as a subfraction of HMG-2 protein by Mathew et al. [1] in the late 1970s. For nearly twenty years, HMGB3 was known as a chicken-specific HMG-2a protein (not to be confused with HMGA2). In 1997, HMGB3′s cDNA was isolated from human and mouse tissues by Wilke et al. [2], and half a year later, it was re-discovered as HMG-4 (not to be confused with HMGB4) by Vaccari et al. [3]. Another protein of the family, named HMG-3, which was also first described in the late 1970s [4] and later identified as a degradation product of the HMG-1 protein [5], has since not been considered a separate protein. At the very end of the 1990s, after a thorough study of the structure and functions of HMG proteins, their new classification was proposed (Table 1) [6,7], according to which HMG-2a (HMG-4) protein was named HMGB3 (not to be confused with HMG-3).

HMGB1-4, also called HMGB proteins, are the most abundant and well-studied proteins of the HMG superfamily. Their characteristic feature is the structural and functional DNA-binding motif known as HMG-box [6,8]. Many transcription factors interact with DNA through functional motifs homologous to the HMG-Boxes of HMGB1 protein [8,9,10,11,12]. There are at least forty-eight highly diverse human proteins containing HMG-boxes [13,14,15]. HMG-box proteins may contain one HMG-box and demonstrate sequence-specific binding to DNA or several HMG-Boxes and show no sequence specificity. For example, single-HMG-Box proteins include the SRY (**s**ex-determining **r**egion of **Y** chromosome) [16,17], the SOX (**s**ry-related HMG b**ox**) subfamily [16,18,19], lymphoid enhancer-binding factor (LEF-1) [20], and chromatin remodeling factors BAF57 and PB1 [13,21,22]. Proteins with multiple HMG-boxes include other proteins alongside HMGB1–4 [23,24,25,26], such as transcriptional factor of RNA-polymerase I UBF [27], drosophila DSP1 protein [28], yeast HMO1/2 protein [29], and mitochondrial factors mtTF1 and ABF2 [13]. A characteristic feature of HMGB1–4 is the non-sequence-specific nature of their DNA recognition [9,10,11,26,30,31], with preferential binding to DNA regions with various structural anomalies [32,33]. HMGB1, HMGB2, and HMGB3 are important cytokines that mediate responses to infection, injury, and inflammation [26,34]. The structure and functions of HMGB1 and HMGB2 proteins have been discussed in other studies [9,10,11,26,35,36]. In this review, we focus on the structural and functional properties of HMGB3—a member of the HMGB family, which also plays an important role in DNA repair, replication, transcription, and recombination [37,38,39]. Currently, there is a limited amount of fragmentary and scattered experimental data regarding HMGB3 in the literature. Therefore, our main goal was to assemble the available data on the structure and known functions of the protein, as well as to compare the gathered data with that on HMGB1 and HMGB2. However, the available information is merely illustrative, and we believe that conclusions made for HMGB1 and HMGB2 (e.g., for their PTMs) should not necessarily be applied to HMGB3. Clearly, conclusions regarding HMGB3 demand careful experimental verification.

## 2. Structure of HMGB3 Protein

**Figure 1:**
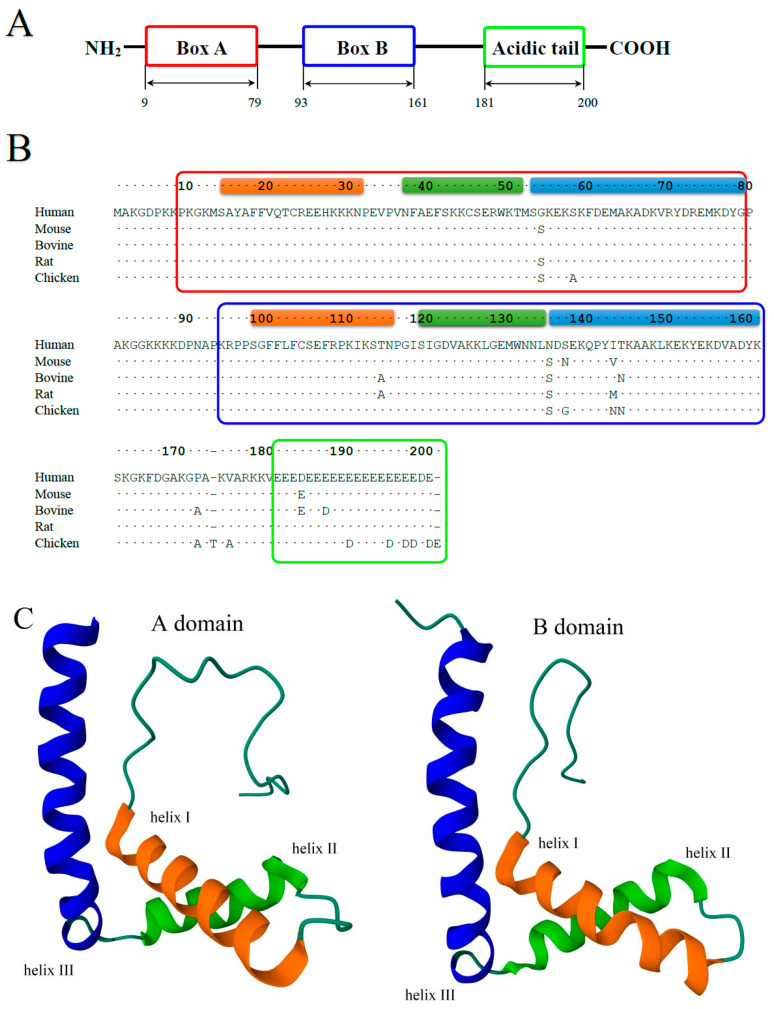
**Structure of HMGB3 protein.** (**A**) Schematic representation of HMGB3 structure. DNA-binding domains (HMG-boxes) are marked with red (N-terminal or A domain) and blue (C-terminal or B domain) frames. The C-terminal acidic tail is marked with the green frame. Numbers indicate the position of the first and last a.a. residue of the corresponding element. (**B**) Sequence alignment of human (UniProt ID O15347), mouse (UniProt ID O54879), bovine (UniProt ID Q32L31), rat (UniProt ID B0BN99), and chicken (UniProt ID P40618) HMGB3 proteins. The HMG-Boxes A and B and C-terminal acidic tails are marked with red, blue, and green frames, respectively (similar to panel (**A**)). The α-Helical regions in each DNA binding domain (HMG-boxes) are marked with orange (helix I), green (helix II), and blue (helix III) blocks. A.a. residues identical to those of human HMGB3 in the corresponding position are indicated with dots. All presented sequences were obtained from the UniProt database [40]. (**C**) Tertiary structure of the HMG-boxes A (**left**) and B (**right**) of human HMGB3 protein. The α-Helical regions in each DNA-binding domain (HMG-box) are orange (helix I), green (helix II), and blue (helix III). The images were obtained using Mol* Viewer Plugin 4.4.1 [41,42] based on the structures of the HMG-box A (PDB ID 2EQZ) and the HMG-box B (PDB ID 2YQI) of human HMGB3 protein available at the RCSB PDB [43].

Compared to the HMGB1 and HMGB2 proteins, HMGB3 has been studied in less detail. According to UniProt [40], the online database of protein structure and functions, general structural information on human (UniProt ID O15347), mouse (UniProt ID O54879), bovine (UniProt ID Q32L31), rat (UniProt ID B0BN99), and chicken HMGB3 proteins (UniProt ID P40618) is available. The amino acid (a.a.) sequences of these proteins are very similar, with a 97.5% identity within mammals and a ~92% identity between mammals and chickens (Figure 1).

**Figure 2:**
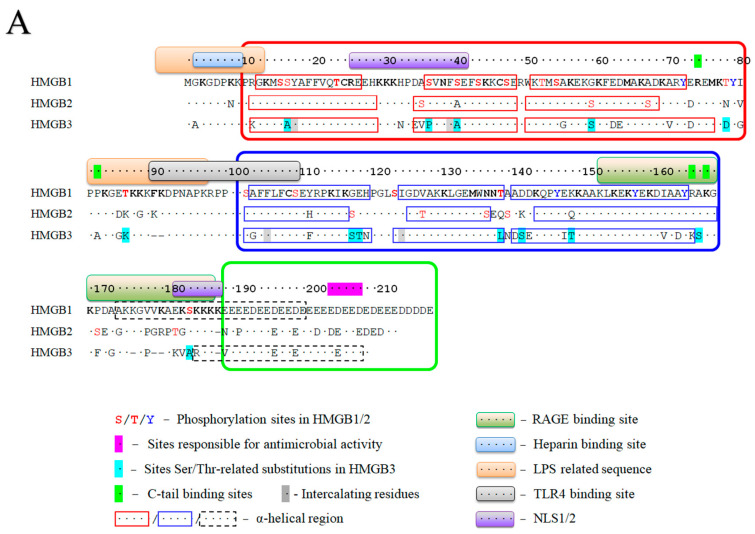

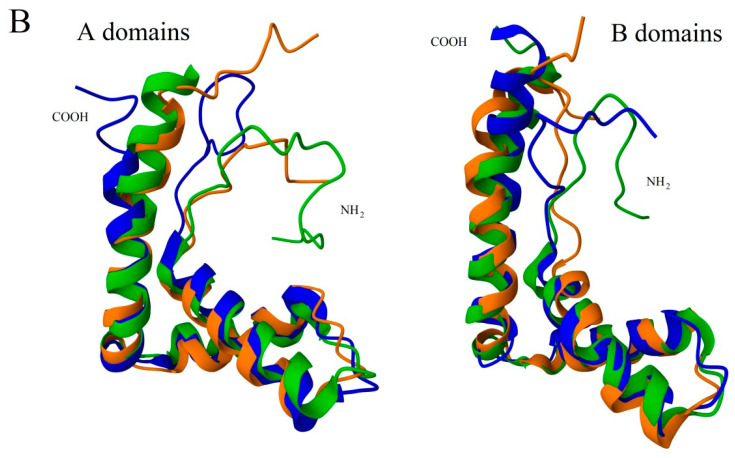
**Structures of human HMGB1–3 proteins.** (**A**) Sequence alignment of the human proteins HMGB1 (215 a.a., UniProt ID P09429), HMGB2 (209 a.a., UniProt ID P26583), and HMGB3 (200 a.a., UniProt ID O15347). The HMG-Boxes A and B and C-terminal acidic tails are marked with red, blue, and green frames, respectively (similar to Figure 1). The α-Helical regions of the sequences are marked with red (within the A domain) and blue (within the B domain) boxes. A.a. residues identical to those of human HMGB1 in the corresponding position are indicated with dots. Positions where PTMs have previously been reported are labeled in bold. All sequences were obtained from the UniProt database [40]. The following abbreviations are used in the legend: TLR4—Toll-like receptor 4; RAGE—receptor for advanced glycation end products; LPS—sequence involved in inhibition of lipopolysaccharide-induced cytokine production; and NLS1/2—nuclear localization sequences 1 and 2. (**B**) Superimposed structures of HMG-Boxes A (**left**) and B (**right**) in rat HMGB1 (orange; PDB IDs 1AAB [40]; 1HME [44]), pig HMGB2 (blue; PDB IDs 1J3X; 1J3D), and human HMGB3 (green; PDB IDs 2EQZ; 2YQI). The images were obtained using Mol* Viewer Plugin 4.4.1 [41] based on the structures available at the RCSB PDB [43,50].

The structural organization of HMGB1-3 proteins is very similar. The proteins consist of a short N-terminal region, two DNA-binding domains (HMG-boxes A and B) connected by a short linker (linker I), and a C-terminal sequence of glutamic and aspartic acid residues (Figure 1A). The tertiary structure of the DNA-binding domains is also very similar in all three HMGB1–3 proteins. According to the NMR data [8,44,45,46] (Figure 1B,C), their HMG-boxes have three α-helical regions, which form two arms of an L-shaped structure. The shorter arm (about 31 Å) is formed by helices I (orange in Figure 1) and II (green), and the longer arm (about 36 Å) is formed by helix III (blue) and the disordered N-terminal fragment (red). The mutual orientation of the arms, which forms an angle of ~70–80°, is fixed by strong hydrophobic interactions between a.a. residues located at the apex of the corner. For all three proteins, the domain structures are remarkably similar, with root mean square deviations (RMSDs) of superposed structures equal to 1.64 (A domains of rat HMGB1 vs. human HMGB3) and 1.50 (A domains of pig HMGB2 vs. human HMGB3). These values are comparable with the deviations of the superposed A domains of rat versus human HMGB1 (RMSD = 1.76). The superposition of the domains demonstrates that the main difference is in the linker region between helices I and II, as well as near the C-terminus of helix III, reflecting higher conformational flexibility of these regions rather than significant structural distinction. Although both HMG-boxes A and B demonstrate DNA-binding activity, their functioning is slightly different. In particular, HMG-box A demonstrates a remarkable ability to recognize pre-bent DNA regions, while HMG-box B is able to bend linear DNA more effectively [47]. When found outside the cell, HMG-box B manifests proinflammatory activity, while HMG-Box A demonstrates anti-inflammatory properties [48]. HMG-box A interacts with the p53 transactivation domain, while no such activity was observed for HMG-box B [49]. A comparison of the HMGB1 and HMGB3 DNA-binding domains (Figure 2) indicates that, although their structures are very similar at all three levels of the structural organization, some differences remain, which can be seen in both A and B domains.

**Figure 3:**
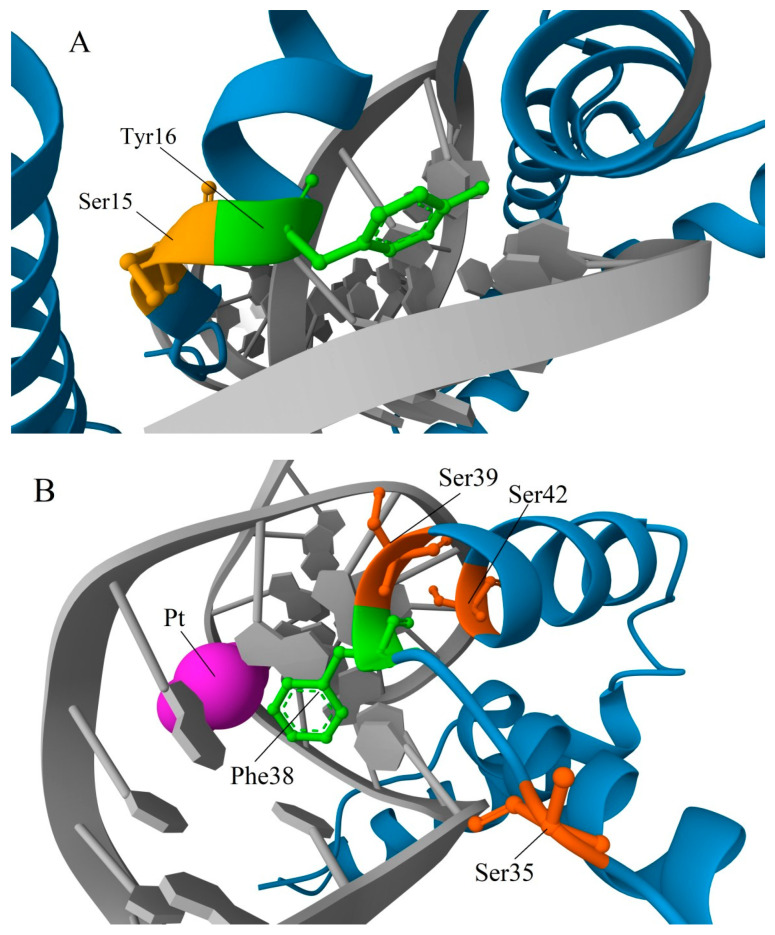
**Effect of Ser/Thr substitutions on the local structure of HMGB3.** Figure represents the local structures of HMGB1/DNA complexes in the vicinity of the substituted Set/Thr sites. (**A**) Locations of Ser15 (orange) and Tyr16 (bright green) in the minor groove of DNA (grey). Protein is colored blue. (**B**) Intercalation of Phe38 (bright green) between the bases of cisplatin (pink)-modified DNA (grey); adjacent Ser39 and Ser42 are marked with orange; Ser35 (orange) located within the linker between helices I and II (blue) in close proximity to the DNA backbone. The images were obtained using Mol* Viewer Plugin 4.4.1 [41] based on the structures available at the RCSB PDB: 4QR9 [53] (**A**) and 1CKT [56] (**B**).

The RMSD values of superposed A and B domains of the same protein for rat HMGB1 and human HMGB3 are 2.66 and 2.64, respectively. In the A domain of HMGB3, all three α-helical regions are slightly longer compared to those in HMGB1. Additionally, several important a.a. substitutions can be observed in the HMG-box A of HMGB3 protein: Ser15 (helix I) and Ser39 (helix II) in HMGB1 are replaced with Ala residues in HMGB3; Ser35 (unordered linker) in HMGB1 is substituted by Pro in HMGB3; and Gly58 (helix III) in HMGB1 is replaced with Ser in HMGB3. Ser and Thr residues are the potential sites of phosphorylation (see Section 3)—a post-translational modification (PTM) with important regulatory functions. Some of the sites listed are located close to other functionally important a.a. residues. In particular, Ser15 is adjacent to Tyr16, which interacts with the DNA bases in the minor groove (Figure 3A) [51,52,53]. Accounting for the α-helical structure of this part of the polypeptide chain, Ser15 is also positioned next to the Phe19, which plays a very important role in the binding of the HMGB domain to cisplatin-modified DNA, partly intercalating between nitrogenous bases [54] (also see Section 4). Ser39 belongs to the nuclear localization sequence 1 (NLS1) [55]. It is also adjacent to the strategically positioned Phe38, which stabilizes complexes of HMGB1 with DNA (Figure 3B) [53,56]. The linker region between helices I and II modulates DNA binding. Further, it belongs to the NLS1 and contains another serine (Ser35) substituted by the Pro35 in HMGB3.

Additionally, there are several important a.a. within the HMG-Box B and linker II region of HMGB3, which are different from those in HMGB1. Gly115 (helix I), Glu116 (helix I), Asp139 (helix III), Lys146 (helix III), and Ala164 (linker II) are substituted by Ser/Thr residues; Thr136 (helix II) is changed to leucine; and Ser181 (linker II) is replaced with Ala residues. All of these Ser/Thr sites are also targets for phosphorylation and, although not involved in direct interaction with DNA, are important for interaction with other proteins, including those containing the RAGE-binding domain (150–183 a.a.) [47] and C-terminal tail of HMGB1 protein (163–165 a.a. and linker II) [57,58]. Thus, one can expect that the functional differences observed for the HMG-boxes A and B in HMGB1 are not so pronounced for the A and B DNA-binding domains in HMGB3.

Human HMGB3 consists of 200 a.a. and has a ~75% sequence identity with human HMGB1 and HMGB2 (Figure 2). One of the major differences in the primary structure of HMGB1–3 is the length of their C-terminal acidic sequences, which decreases gradually from 30 a.a. in HMGB1 to 22 and 20 in HMGB2 and HMGB3, respectively, while the C-terminal acidic sequence is completely missing in HMGB4 [23]. Such negatively charged tails are not a unique feature of HMGB proteins and can be found in other families, including HMGA [59,60]. These tails regulate protein functions mainly through interaction with nucleic acids and other proteins. The difference in charge between the proteins’ positively charged DNA-binding motifs and these negatively charged tails helps to induce structural distortions in DNA upon binding [60,61,62]. Thus, the differences in the length of this region may have important functional consequences. It has been demonstrated that the negatively charged tail of HMGB1 interacts with DNA-binding domains and that positively charged linker regions regulate the transition between two conformational states of the protein [57,58,63,64]. One of these states is conformationally stable but functionally inactive (“closed”), while the other corresponds to a more flexible and functionally active (“open”) conformation. Additionally, it has been demonstrated that the acidic tail of HMGB1 modulates the antibacterial and anticancer properties of the protein [65,66].

Alongside a number of the dicarboxylic a.a. residues, the C-terminal tails of the HMGB1–3 proteins demonstrate some sequence variations (Figure 2), which might have structural significance. Recently, it was demonstrated that Asp and Glu residues have different effects on the structural stability of the polypeptide chains. The study revealed differential effects of these a.a. in intrinsically disordered regions (IDRs) in proteins, and their substitutions with other a.a. may contribute to structural and functional diversity. It has been demonstrated that Glu residues can support increased helicity, which is important for folding and binding, while Asp residues support extended structures [67].

**Figure 4:**
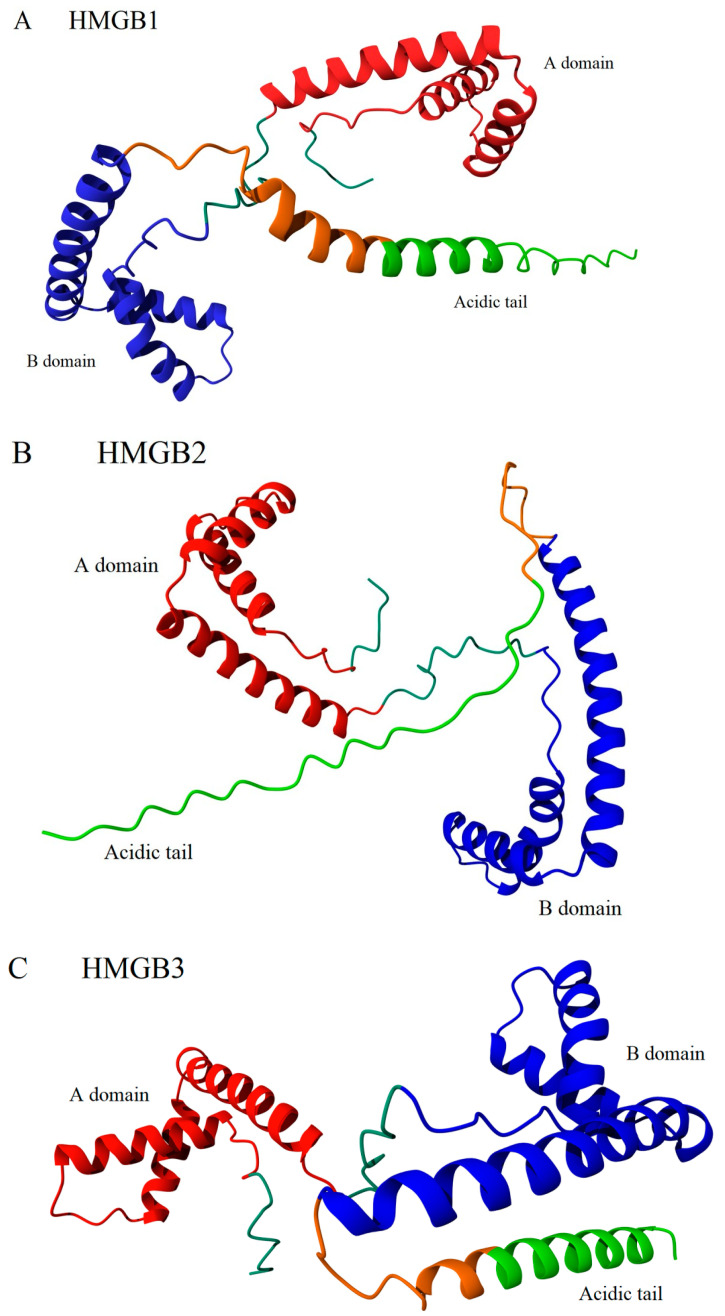
**Three-dimensional models of full-length human HMGB1–3 proteins predicted by AlphaFold 2.** Structures predicted for human (**A**) HMGB1 (UniProt ID P09429), (**B**) HMGB2 (UniProt ID P26583), and (**C**) HMGB3 (UniProt ID O15347) using AlphaFold 2 [42]. A (N-terminal) and B (C-terminal) DNA-binding domains (HMG-boxes) are colored in red and blue, respectively; C-terminal acidic tails are green; and the linker between B domain and C-tail (linker II) is orange.

The acidic tail of HMGB1 is considered an example of an IDR. The interaction between the positively charged a.a. residues in the linker region adjacent and the negatively charged groups of the C-terminal domain stabilizes the conformation of the glutamate-rich polypeptide chain closest to the linker. However, regular inclusions of Asp residues lead to rapid destabilization of the secondary structure of the Glu-enriched sequence. Interestingly, in HMGB3, most of the Asp residues in the tail region are substituted by Glu residues, which form a long poly-Glu sequence that is more favorable for the formation of the regular secondary structures. In contrast, in HMGB2, the linker sequence and the adjacent part of the acidic tail contain three proline residues, eliminating any possibility for a helix formation in this region. Although the structures of many individual HMGB domains have been elucidated, experimental data on the structure of any of the full-length HMGB1–3 proteins remain missing. Indirect confirmation of the structural significance of the outlined differences in sequences of HMGB1–3 is also provided by the results of the tertiary structure prediction performed by AlphaFold 2 [42,68,69]. According to these calculations, the a.a. substitutions within the C-terminal domain and the adjacent linker region have led to significant differences in the secondary structure of the acidic tails of all three proteins (Figure 4). The tail is expected to be completely disordered in HMGB2, partly helical in HMGB1, and predominantly α-helical in HMGB3.

The described differences in the structure of the negatively charged C-terminal tails in HMGB1–3 proteins are expected to have a significant impact on their interactions with nucleic acids and other proteins.

## 3. Post-Translational Modifications of Human HMGB3

Post-translational modifications (PTMs) of proteins affect conformation, stability, sub-cellular localization, and intermolecular interactions. Like any other protein, HMGB3 is subject to various post-translational modifications. However, in contrast to HMGB1 and HMGB2, there is very little experimental data regarding the PTMs of HMGB3. Therefore, the majority of the PTMs attributed to HMGB3 are, in fact, predicted by analogy with the HMGB1 and HMGB2 proteins. The main PTMs in HMGB1–3 proteins are acetylation, phosphorylation, and methylation, which, along with the redox status, strongly affect their functions [9,11,35,36,55,70,71,72,73].

### 3.1. Acetylation

Acetylation of Lys residues is one of the common modifications reported for HMGB1 and HMGB2 proteins (Figure 2) [26,55,70,73,74]. Acetylation—identified earlier within the disordered regions flanking DNA-binding domains—affects the binding sites of heparin and several proteins such as RAGE, TLR4, and IL. (Figure 2) [75,76,77]. In the case of HMGB1, Lys3 acetylation has been shown to increase the binding affinity of the protein to DNA [78]. Similar to HMGB1 and HMGB2, the cellular localization of HMGB3 is likely affected by the acetylation of a.a. residues within two nuclear localization sequences (NLS1 and NLS2). It was shown that the acetylation of HMGB1 Lys residues in the NLS regions is directly related to the localization of the protein in the nucleus or cytosol and its participation in the transmission of anti-inflammatory response signals. Simultaneously, acetylation of HMGB1 lysines within the NLS regions 27–43 a.a. (NLS1) and 178–184 a.a. (NLS2) were associated only with active secretion and shown to be atypical for the passively secreted protein [55]. The NLS1 region is located within HMG-Box A of the protein. Comparison of the a.a. sequences of HMGB1–3 (Figure 2) showed that the region corresponding to NLS1 is similar for all three proteins. As for the NLS2 region, the picture is somewhat different. NLS2 is located at the edge of the B domain, corresponding to the a.a. 173–179 in HMGB3.

**Figure 5:**
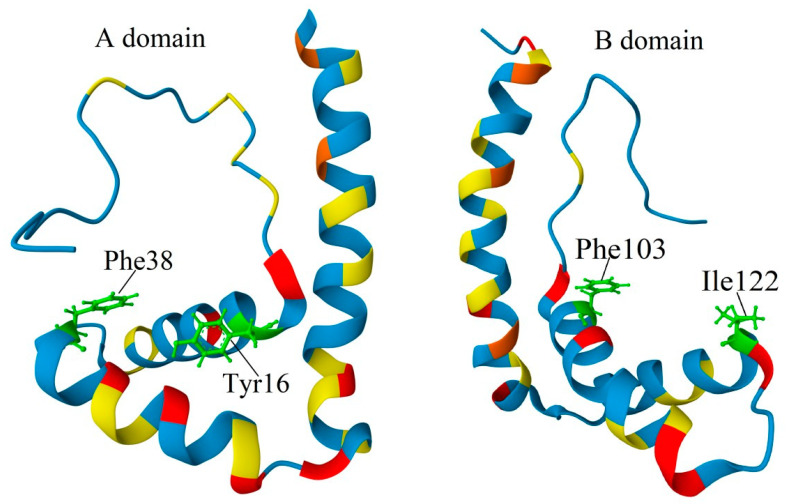
**Post-translational modifications of HMGB3 protein**. Figure 5 demonstrates the location of the potential sites of Lys acetylation (yellow), Ser/Thr phosphorylation (red), and Tyr phosphorylation (orange) in human HMGB3 DNA-binding domains A (**left panel**) and B (**right panel**). Strategically positioned a.a. residues Tyr16/Phe38 (A domain) and Phe103/Ile122 (B domain) are colored in green. For a complete map of currently known PTM sites, see Figure 2. The images were obtained using Mol* Viewer Plugin 4.4.1 [41] based on the structures available at the RCSB PDB: 2EQZ (domain A) and 2YQI (domain B) [43,50].

Although potential acetylation sites are numerous in both DNA-binding domains in HMGB3, their location is somewhat different (Figure 5). There are eleven potentially acetylated Lys residues in the A domain, and only four of them, namely Lys12, 43, 50, and 68, are directly involved in DNA binding. The other seven Lys residues are located on the outer surface of the DNA protein complex, have no direct interaction with DNA, and are probably involved in interaction with other proteins. A similar situation occurs in the B domain, where only four Lys residues (Lys94, 112, 125, and 150) participate in DNA binding.

Another aspect that we would like to highlight is the preferential binding of proteins of the HMGB family to DNA regions with various structural abnormalities. Using HMGB1 as an example, it was demonstrated that acetylation affects its ability to bind to platinum adducts on DNA (Figure 3B) and to DNA regions damaged by UV light [79]. Based on the high homology in a.a. sequences (especially within the A domain) and the similarity of the tertiary structures of their DNA-binding domains, one can assume that acetylation of Lys residues in HMGB2 and HMGB3 also affects their affinity to DNA regions with “distorted” structures, as well as DNA replication and repair [78].

### 3.2. Methylation

It was reported earlier that Lys43 of HMGB1 was methylated in neutrophils [74]. The Lys in this position is found in many HMG-box proteins, including HMGB3. When complexed with DNA, Lys43, positioned on the outer surface of the complex close to the sugar-phosphate backbone, is able to establish electrostatic interactions with the adjacent phosphate group of DNA [51,53,56,80]. Once methylated, Lys43 does not interact with the phosphates anymore, thereby weakening the interaction between the DNA and protein, which in turn might help HMGB1 to leave the nuclear space and migrate into the cytosol. As Lys43 is in exactly the same environment in HMGB3 as in HMGB1 protein (FS**K**KCSERWK), one can expect that Lys43 in HMGB3 might also undergo methylation, facilitating the translocation of the protein from the nucleus to the cytoplasm [74].

As mentioned, tumor cells are characterized by a high level of HMGB3 expression. Gong et al. [81] analyzed HMGB3 methylation in various tumors with high HMGB3 expression, revealing that the majority of samples studied were characterized by hypomethylation of HMGB3. In contrast, hypermethylation of this protein was observed in the cells with low levels of HMGB3 expression. On the other hand, HMGB3 can participate in tumor development, migration, and proliferation of tumor cells through the WNT/β-catenin signaling pathway. Zheng et al. [82] suggested that breast cancer cells were characterized by hypomethylation of HMGB3 and HOX transcript antisense RNA (HOTAIR).

Although experimental results have demonstrated the importance of methylation in regulating HMGB3 functions, its specific mechanisms are yet to be unraveled.

### 3.3. Phosphorylation

Phosphorylation of HMGB proteins by calcium–phospholipid-dependent protein kinase was first demonstrated by Ramachandran in 1984 [83]. For HMGB1, calcium/calmodulin-dependent kinase IV (CaMK IV) has been shown to be involved in HMGB1 release induced by lipopolysaccharides (LPS) through phosphorylation of Ser residues [71]. Much later, it was found that HMGB proteins can be phosphorylated with the help of other kinases: PKC (protein kinase C), CKII (Casein Kinase II), and CDC2 (cell-cycle-dependent kinase) kinase [71,79]. Regardless of the type of kinase involved in protein phosphorylation, this process is directly related to the concentration of calcium ions in the cell.

Phosphorylation, as well as acetylation and methylation, influences the sub-cellular localization of HMGB3 and additionally regulates the interaction between the protein and DNA [26]. Several potential phosphorylation sites can be identified in HMGB3. First, there are fifteen Ser/Thr residues, which can be phosphorylated (Figure 5). Interestingly, all of them are located within the helical regions of DNA-binding domains (seven in the A domain; eight in the B domain), which is significantly different from the distribution of these a.a. residues in HMGB1 and HMGB2 proteins. In HMGB1, most of the Ser/Thr residues are found in the A domain (ten out of sixteen), four are located in the B domain, and one is located in each linker region. Moreover, there are no Ser/Thr sites in helices III; instead, they are located within helices I and II, which are involved in DNA binding in both HMGB domains of HMGB1. In HMGB2, there are thirteen Ser/Thr residues in the A domain, seven in the B domain (none in helix III), and three in the linker II region. As previously mentioned, the functioning of the HMG-boxes A and B in HMGB1 and HMGB2 is somewhat different, and varied phosphorylation patterns might thus be important in the regulation of their DNA-binding activity, while phosphorylation sites within the NLS regions might affect the nuclear/cytoplasmic distribution of the protein. However, in the case of HMGB3, the only phosphorylation site found in the NLS sequences is Ser42, located at the edge of NLS1, while the phosphorylation patterns in the HMG-boxes A and B appear quite similar. Although there is a lack of Ser/Thr sites in helices III in HMGB1 and HMGB2, there are several sites of Tyr phosphorylation exclusively in helices III [73], indicating that the functioning of the proteins requires two different mechanisms of the phosphorylation of DNA-binding domains: one for helices I/II and the other for helix III. Furthermore, HMGB3 retains all potentially phosphorylated Tyr residues in helices III but also has several Ser/Thr sites in these regions, implying that the regulation of its interactions with nucleic acids and other proteins might be more complex than could be expected based on the similarity with HMGB1 and HMGB2.

### 3.4. Glycosylation

It was reported earlier that Asn glycosylation is yet another PTM that determines the intracellular localization of HMGB1 and its export from the cell [84]. N-glycosylation of Asn residues occurs when they are located within a consensus motif of Asn-Xxx-Ser/Thr (Xxx is any a.a. except proline). Two sites of glycosylation were reported in HMGB1: Asn37 and Asn134, both located within helices II of HMG-Box A and B, respectively (Figure 2). Kim et al. [84] demonstrated that, in HMGB1, glycosylation of these Asn residues reduces the affinity of the protein to DNA and enhances its association with the nuclear export protein CRM1. The latter is essential for HMGB1 to exit the nucleus into the cytoplasm and then move into extracellular space [55].

Comparison of the a.a. sequences of HMGB1 and HMGB3 shows that despite the fact that both mentioned Asn residues (Asn37/Asn134) are also present in HMGB3 (Asn37/Asn132, respectively), both consensus sequences are disrupted due to the replacement of Ser39 and Thr136 (HMGB1) with Ala39 and Leu134 (HMGB3), respectively (Figure 2). However, multiple a.a. substitutions in positions 134–138 formed a new consensus sequence, with a potential glycosylation site of Asn135 located between helices II and III in the HMG-Box B of HMGB3. Thus, despite the lack of experimental evidence, one can anticipate glycosylation of Asn135 in HMGB3.

### 3.5. Ubiquitination

Ubiquitination is a post-translational modification of proteins involved in many cellular processes, including protein degradation, protein quality control, intracellular trafficking, mRNA translation, endocytosis, DNA damage response, and intracellular signaling [85]. Ubiquitination occurs via the covalent binding of the C-terminal glycine of a ubiquitin, a 76 a.a. protein, to a lysine residue on a substrate protein via an enzymatic machinery composed of ubiquitin-activating enzymes (E1s), ubiquitin-conjugating enzymes (E2s), and ubiquitin ligases (E3s). Ubiquitin can be attached to a target protein as a single molecule (monoubiquitination) or multiple molecules (polyubiquitination). In a simplistic manner, monoubiquitination is thought to be largely associated with chromatin regulation, protein sorting, and trafficking [86,87,88], whereas polyubiquitination has been linked to protein signaling and degradation by the ubiquitin–proteasome system and autophagy [89,90].

The ubiquitination of HMGB3 is still poorly understood. BRCA1, as a RING domain of E3 ubiquitin ligase, was shown to ubiquitinate HMGB3 and accelerate the proteasome degradation of HMGB3 in human thyroid cell lines [91]. Moreover, SYT7 was found to interact with BRCA1 and negatively regulate the ubiquitination of HMGB3, thereby stabilizing this protein and promoting thyroid cancer progression [91]. According to the UbiBrowser 1.0 [92], there are forty-eight E3 ubiquitin ligases predicted for human HMGB3 as a substrate, forty-six of which are also predicted for human HMGB1. Among the latter forty-six proteins is RNF125, for which HMGB1 has been experimentally identified as a substrate [93]. RNF125 interacts with the HMG-Box B of HMGB1 and induces degradation via the ubiquitin–proteasome system, reducing autophagy and oxidative stress in human bronchial epithelial cells. E3 ubiquitin ligase RNF186 has also been found to ubiquitinate cytoplasmic HMGB1, leading to its subsequent proteasomal degradation [94]. Another E3 ligase, CHIP, can interact with HMGB1 and promote its ubiquitinated degradation, thereby inhibiting aerobic glycolysis and the progression of endometriosis [95]. As HMGB3 is generally accepted to be structurally 80% identical to HMGB1 at the a.a. level and consists of three specific functional regions, we can speculate that these E3 ligases could also ubiquitinate HMGB3. According to the web-based bioinformatics resource PhosphoSite, there are ten ubiquitination sites of human HMGB3: Lys30, 43, 59, 112, 125, 126, 139, 145, 161, and 170 [96]. All these ubiquitination sites have been identified in large-scale proteomic studies, and the functional significance of each of these sites has not been studied yet. It is important to note that all ubiquitination sites in HMGB3, except Lys161 and 170, are also ubiquitination sites in HMGB1 and HMGB2 proteins. Moreover, more than half of the human HMGB3 ubiquitination sites are also acetylated or succinylated (Lys43, 59, 139, 145, 161, and 170). Therefore, HMGB3 transport may be regulated via crosstalk between acetylation and ubiquitination. Notably, lysine is the most frequently post-translationally modified residue of all proteinogenic a.a. and is a target for ubiquitination, sumoylation, acetylation, succinylation, and methylation, among others, which suggests Lys PTMs may be the most significant means through which signaling pathways modify protein behavior in cells [97].

Recently, exosomal HMGB3 has been found to be a proinflammatory modulator of silica-induced inflammation, promoting the inflammatory response and recruitment of monocytes/macrophages by regulating the activation of the STAT3/MAPK/NF-κB/CCR2 pathways [98]. In another study, HMGB3 was shown to be enriched in glioma cell-secreted exosomes and confer the activation of NLRP3 inflammasome and pyroptosis in tumor-associated macrophages [99]. We can suggest a role of the ubiquitination of in-tagging HMGB3 for delivery to extracellular vesicles because the ubiquitination is considered a modification sufficient for direct trafficking of soluble proteins within the phagocytic/endocytic network to exosomes [100].

### 3.6. Redox State of HMGB3

Cys residues in positions 23, 45, and 104 (106 in HMGB1/2) in HMGB1–3 proteins were found in both oxidized and reduced states [35]. Cys23 and Cys45 were conserved in these proteins [101] and located in the DNA-binding domain A near a.a. that influence the DNA-binding properties of both HMGB3 and its structurally similar homologs HMGB1 and HMGB2 [56]. The formation of a disulfide bond between Cys23 and Cys45 results in different states of oxidation (as in the case of HMGB1 protein [11]): a fully reduced protein (all three cysteine residues are reduced HMGB3-C23^H^C45^H^C104^H^), a disulfide form (HMGB3-C23-C45C104^H^), and a sulfonyl form (all cysteine residues are oxidized HMGB3-C23^SO^C45^SO^C104^SO^) [102]. All proteins in the HMGB1–3 group are characterized by conservative cysteine in the N-terminal part of the HMGB B domain in position 106 of HMGB1 and HMGB2 proteins [103] and in position 104 of HMGB3. Based on the results of the HMGB1 studies, we may expect that the redox state of the cysteines determines the localization of the proteins in the cellular/extracellular space and, consequently, their functions [9,11,35,36,70]. In the case of the HMGB1 protein, it was reported that the oxidation of Cys23 and Cys45 (the third cysteine residue remains in a reduced form) resulted in the release of the HMGB1 protein into the cytoplasm [35,70] and into the extracellular space [35], where it acts as an alarmin. Alarmins play an important role, particularly in enhancing the body’s inflammatory response to SARS-CoV-2 infection [75,104,105]. The lack of oxidation in Cys45 and Cys105 in HMGB1 may have a significant impact on HMGB1 binding to DNA [106], as well as on interaction with non-canonical DNA structures [10,11,26,32].

It should be noted that recent studies of breast cancer have shown that, along with PTMs characteristic of almost all protein molecules, there is a missense mutation in HMGB3 that involves the replacement of Lys with Asn in positions 30 and 155 [107].

Thus, all described HMGB3 PTMs are likely to affect functions and the distribution of this protein in the cellular/intercellular space.

## 4. Molecular Partners of HMGB3

Knowledge of the protein structure is undoubtedly very important for understanding its functions. However, it is the interaction between the protein and other biological molecules that allows us to fully characterize the diversity of its functions and helps to assign new functions to molecules with unknown roles. Although the pattern of interactions between HMGB3 and DNA and other proteins has not yet been sufficiently studied, it is possible to conclude that there is a wide variety of HMGB3′s molecular partners, indicating the involvement of HMGB3 in many processes in the cell, such as DNA replication, transcription and translation, signal regulation transmission, and cellular pathways of degradation. Here, we attempted to describe what we judge to be the most significant interactions discovered to date.

### 4.1. DNA

HMGB1–3 are primarily nuclear proteins, and their release into the cytoplasm and then into the extracellular space can occur only under certain conditions (oxidation of cysteine residues, variety of PTMs, etc.). The main function of these proteins in the nucleus is to act as DNA chaperones: modulation of chromosome destabilization, replication, transcription, DNA repair, and telomerase maintenance. In the cytoplasm and outside the cell, these proteins function as cytokines or chemokines and cause an immune response and inflammation [9,11,35,108].

All currently known HMG-box proteins are capable of binding DNA via their HMG-boxes [9,10,11,26], having demonstrated the ability to bend DNA upon binding [9,11,26,30,109,110] or recognize pre-bent regions of the double helix, including those induced by the antitumor drug cisplatin [32,111,112]. Additionally, HMG-box proteins demonstrate high affinity to cruciform and four-way junction DNA [26,33,113]. For the HMG-boxes of the HMGB1 protein, it was shown that their DNA-binding properties are somewhat different despite their structural similarity. As mentioned, HMG-box A readily recognizes pre-bent DNA regions, while HMG-box B bends linear DNA on its own [47]. Although no experimental structures of HMGB3/DNA complexes have been reported to date, some predictions can be made based on the similarity to previously solved structures for other HMGB proteins as well as simulation using AlphaFold 3 [114].

**Figure 6:**
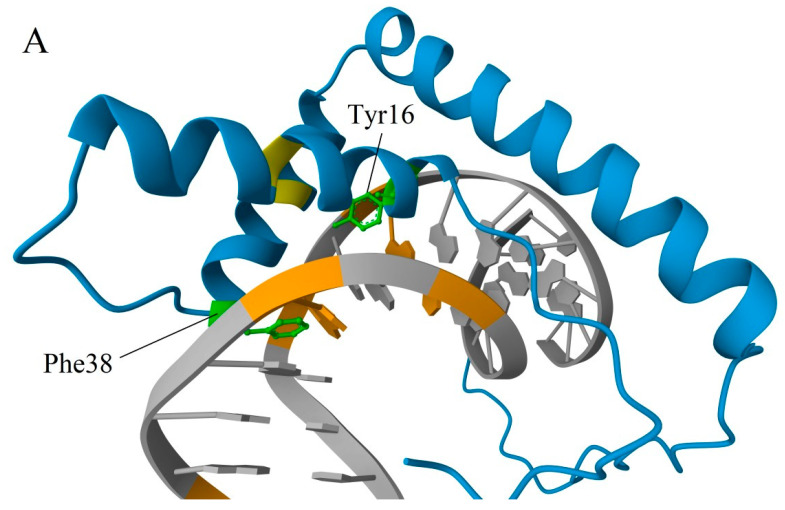

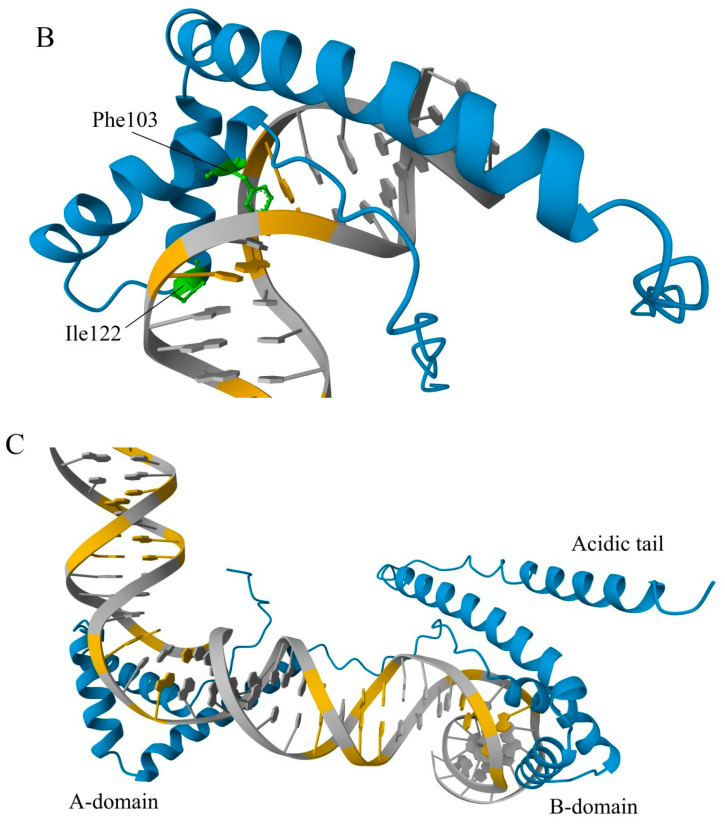
**Three-dimensional models of HMGB3/DNA complexes.** (**A**) Complex between 26-mer dsDNA and the A domain in human HMGB3. Yellow—Cys23/Cys45; green—intercalating residues. (**B**) Complex between 26-mer dsDNA and the B domain of human HMGB3. Green—intercalating residues. (**C**) Complex between 43-mer dsDNA and human HMGB3. The sequence of 26-mer dsDNA (one of the two complementary strands) is the following: CCGCGCCTGTGGGATCTGCATGCCCC. The sequence of the 43-mer dsDNA (one of the two complementary strands) is the following: GGGGCATGCAGATCCCACAGGCGCGGAGATCCCACAGGCGCGG. Grey—DNA strands; orange—A–T pairs in DNA; blue—protein. The images were obtained using Mol* Viewer [41] based on the structures simulated by AlphaFold 3 [114].

Both HMG-boxes interact with DNA in the minor groove, anchored by side chains of strategically positioned a.a. residues located at the N-termini of helices I and II. In the A domain, Tyr16/Phe38 are the a.a. residues located at the N-termini of helices I and II, while Phe103/Ile122 are the residues in the B domain (Figure 5). In this case, the aromatic rings Phe38 and Phe103 partially intercalate between DNA base pairs (Figure 6). The crossed configuration of two α-helices (that can be additionally stabilized in the A domain by the S-S bond Cys23-Cys45) forms an angle close to 110° on the interaction surface, which induces a corresponding bend of the double helix upon binding to DNA.

According to the model obtained by AlphaFold 3 (Figure 6C), when both HMGB domains in HMGB3 interact with DNA, two bends of approximately 110° each are induced in the double helix. However, the bends are formed in mutually perpendicular planes, so the U-turn type structure, previously observed for some HMG-box proteins [115,116], is not formed here. The acidic tail of the protein is located along helix III of the B domain, establishing electrostatic interactions between the positively charged lysine residues of helix III and the negatively charged glutamic acid residues of the C-terminal domain.

All HMGB proteins bend DNA, thereby facilitating the recruitment of transcription factors and other class proteins [10,26,30,117]. In addition, HMGBs recognize and preferentially bind DNA with various structural abnormalities [9,11,56,118], including cisplatin-modified DNA. Interestingly, HMGB3 is involved in chemotherapy resistance in some tumors [39,119,120,121,122]. Li et al. [123] showed that in cervical cancer, HMGB3 specifically binds to the promoter region of human telomerase reverse transcriptase (hTERT) and induces hTERT expression, leading to radioresistance in tumor cells. hTERT promotes the repair of damaged DNA regions, which can cause cell cycle arrest and/or apoptosis, thereby increasing the resistance of cancer cells to chemotherapy or radiation therapy. HMGB3 binds platinum adducts to DNA, which promotes the initiation of nucleotide excision repair, inhibits tumor cell apoptosis, and stimulates proliferation [124,125]. Overexpression of HMGB3 can inhibit DNA bending, thereby interfering with the recruitment of transcription factors [39]. Targeted depletion of HMGB3 sensitizes chemoresistant ovarian cancer cells to cisplatin by suppressing the ATR/CHK1/p-CHK1 DNA damage signaling pathway [119]. HMGB3 was found to target miR-27b—the event associated with tamoxifen resistance in breast cancer [121]. However, the particular functions of HMGB3 in the development of resistance to chemo- and radiotherapies in various types of cancer remain unclear.

### 4.2. RNA

To date, a sufficient amount of experimental data has been collected to indicate that, in addition to DNA, many (if not all) HMG-box proteins are capable of binding to RN (see detailed review [14] and references therein), including HMGB3. Yan et al. recently identified HMGB3 among RNA-binding proteins in the nucleus and cytoplasm [126]. Earlier, Yanai et al. [34] demonstrated that HMGB3 binds immunogenic nucleic acids, including RNA, facilitating their binding to pattern recognition receptors, such as TLRs. Khoury et al. [127] showed that HMGB3 binds to structurally specific sites of HIV-1 tat mRNA (Tat: trans-activator of transcription), controlling tat mRNA processing and translation. Furthermore, HMGB3 has been found in nucleoli and stress granules [14]. Nucleoli represent cellular centers of rRNA assembly and processing, while stress granules represent cytoplasmic ribonucleoprotein (RNP) complexes, which are formed in response to cellular stress.

As described, one of the key properties of HMG-boxes is their ability to bind DNA in the B conformation within the minor groove, resulting in strong bending of the double helix. Such bending is one of the important factors determining the functionality of HMG-box proteins in chromatin. Double-stranded RNA (dsRNA) is in the A form, which differs significantly in its geometry from B-DNA. In this regard, it is extremely interesting to ascertain if the HMGB domains are also able to bend the RNA in double-helical form. Currently, there are no experimentally determined structures of HMG-boxes with RNA. However, it was possible to obtain some information about the possible structure of HMGB3-RNA complexes based on analysis of the models generated using AlphaFold 3 [114], which showed very good results in modeling complexes of HMG-box proteins with DNA.

**Figure 7:**
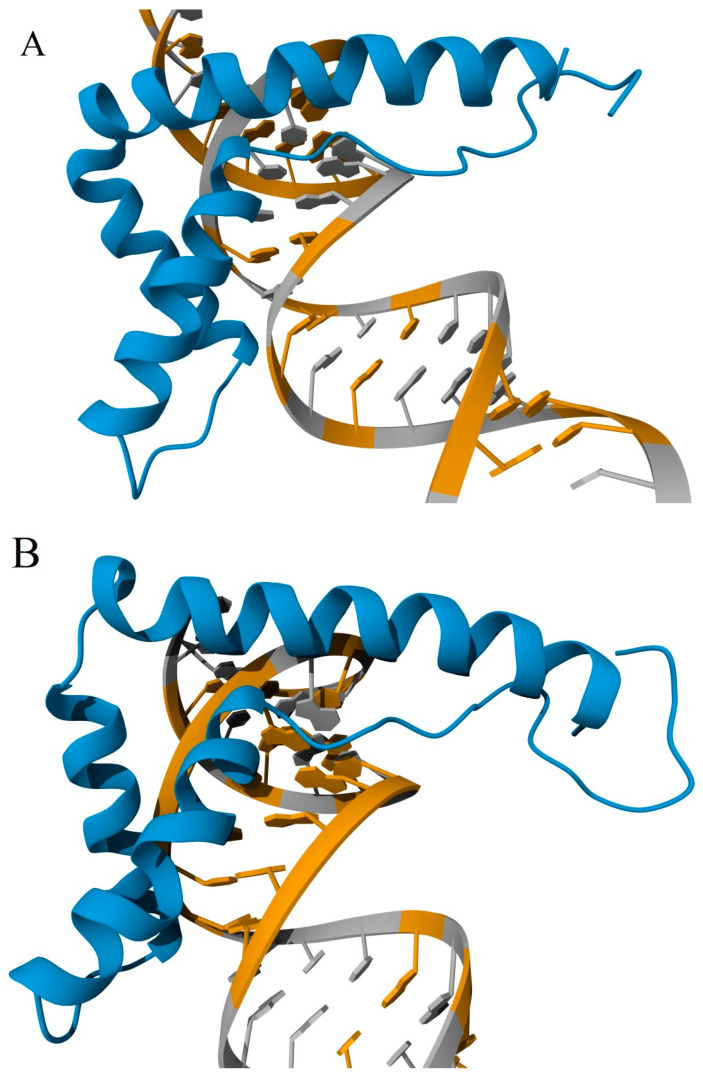

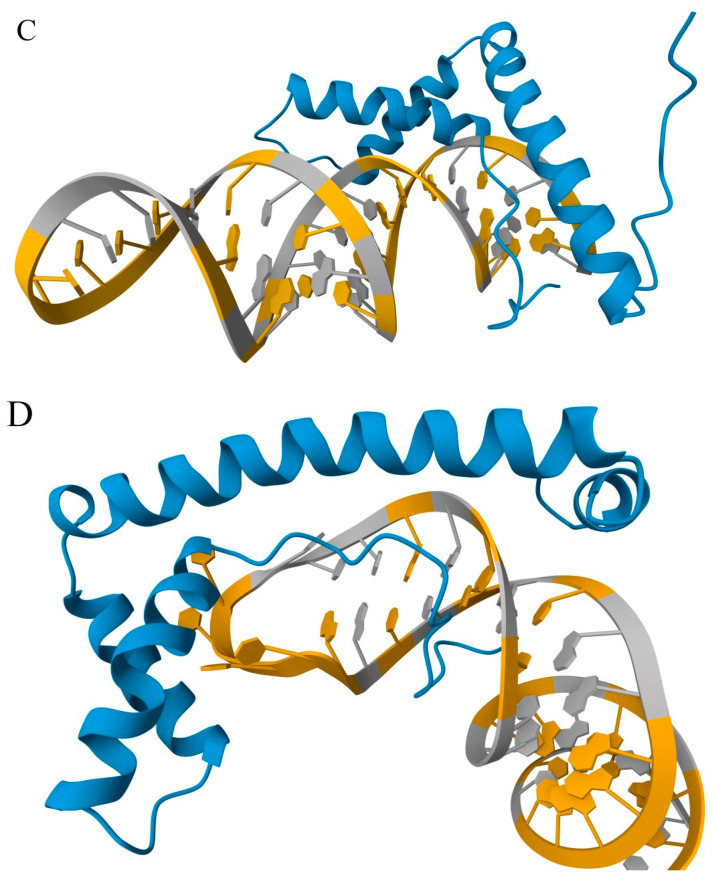
**Three-dimensional models of the complexes between RNA and individual DNA-binding domains of HMGB3.** (**A**) Complex between dsRNA and the A domain of human HMGB3. (**B**) Complex between dsRNA and the B domain of human HMGB3. (**C**) Complex between stem–loop RNA and the A domain of human HMGB3. (**D**) Complex between stem–loop RNA and the B domain of human HMGB3. Grey—RNA strands; orange—A/U nucleotides in RNA; blue—protein. The sequence of 32-mer dsRNA (one of the two complementary strands) is the following: CUAGAGAUCCCUCAGACCCUUUUAGUCUGUGG. The sequence of the 43-mer RNA strand, forming stem–loop, is the following: CUAGAGAUCCCUCAGACCCUUUUAGUCUGUGGAAAAUCUCUAG. The images were obtained using Mol* Viewer [41] based on the structures simulated by AlphaFold 3 [114].

The 3D models obtained using AlphaFold 3 (Figure 7) suggest that the interactions between both DNA-binding domains and dsRNA take place at the surface corresponding to the minor groove of DNA, which is readily available for the interactions in the A form of the double helix. Similar to DNA-protein complexes, the HMG-boxes interact with dsRNA through the same a.a. residues in helices I and II. The structure suggests slight intercalation between the base pairs of RNA and Phe38 in the A domain and Phe101 in the B domain. However, in contrast to HMGB-DNA complexes, no significant bending of the RNA double helix is observed in complexes with either domain. Surprisingly, the B domain demonstrated preferential binding to the AU-rich region of the studied RNA sequence (Figure 7B). When the stem–loop structure was used in the simulation, the B domain interacted with the AT-rich loop (Figure 7D). Although the observation of these loop-binding properties is supported by the mentioned experimental studies on HMGB3-RNA interactions, more research is required to understand the structure and functions of the HMGB-RNA complexes.

**Figure 8:**
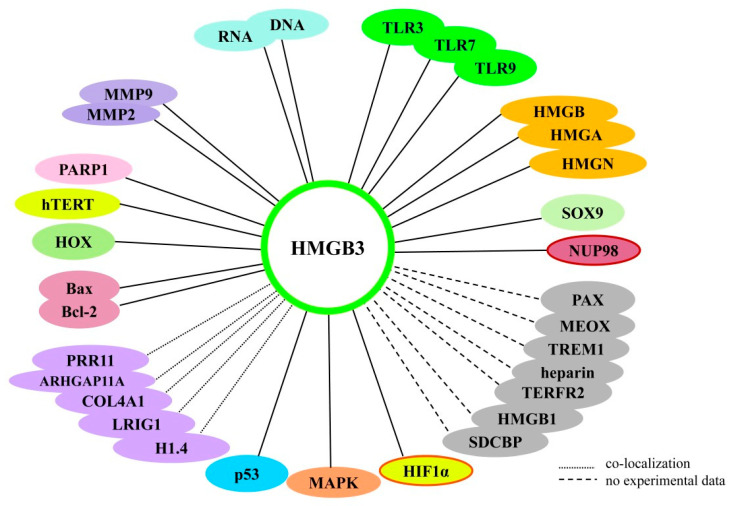
**Protein–protein interaction network for human HMGB3 protein.** The figure was composed based on earlier published data (Table 2) and the IntAct database [128]. Some HMGB3 partners are grouped according to their properties: light blue—nucleic acids; green—Toll-like receptors (TLR); orange—proteins of the high-mobility group (HMG) family; light-lilac—matrix metalloproteinases (MMP); pink-beige—protein factors of apoptosis (Bcl-2/Bax); purple—co-localized proteins; grey—potential partners.

Although there are many similarities in the nucleic acid-binding properties of HMGB1, HMGB2, and HMGB3, their protein partners are significantly different. Using various methods, a number of proteins interacting with HMGB3 were identified (Table 2). Figure 8 shows the interactome generated from the IntAct database, illustrating possible interaction between human HMGB3 protein and some protein components of cells, while Table 2 summarizes the major partners of HMGB3 protein. We will examine HMGB3′s interaction with some of these major partners in more detail in the following sections.

### 4.3. p53

As mentioned, all HMGB proteins have been closely associated with the emergence, proliferation, and metastasis of tumor cells. One of the proteins interacting with HMG-boxes is the tumor suppressor p53. It has been shown that p53′s interaction with DNA requires participation on the part of the HMGB proteins, whereby HMGB1–3 proteins bend DNA, strongly facilitating p53 recruitment in doing so, and then leave the complex [9,11,129,130]. Impaired binding of HMGB1–3 to DNA reduces p53 recruitment to DNA, affecting the antitumor activity of p53 [39]. However, a closer look at the structure of the HMGB1-p53 complex [49] reveals that the p53 interaction involves several specific a.a. residues in the A domain. In particular, Ser34 and Ser38 in HMGB1 interact with Thr55 and Asp41 in p53. In HMGB3, the Ser residues are substituted with Pro and Ala, respectively (Figure 2). This implies that the interaction interface must be formed differently if the interaction between HMGB3 and p53 occurs.

### 4.4. HOX Proteins

It has been proposed that HMGB3 can interact with HOX proteins in primitive blood cells. Increased expression of HOXB6 [131,132], HOXB7 [133], and HOXB8 [134] has been shown to impede myeloid differentiation in hematopoietic cell lines. Furthermore, Nemeth et al. [135] showed that overexpression of HMGB3 can enhance the recruitment of these HOX proteins to their cognate binding sites. On the other hand, HOXB6 expression has been shown to play an important role in erythropoiesis and the regulation of early erythrocyte progenitors [136]. Therefore, the co-expression of HMGB3 and HOXB6 indicates a joint role of these proteins in erythropoiesis.

### 4.5. PARP1

Experimental data, including proteomic analysis based on mass spectrometry, showed a direct interaction between HMGB3 and PARP1 (poly-ADP-ribose polymerase 1) [137]. PARP1 plays a critical role in DNA repair processes through its poly (ADP-ribosylation) (PARylation) activity; specifically, it promotes the repair of damaged bases and single-stranded DNA breaks via modulation of chromatin structure and DNA repair factor recruitment [138]. PARP1 uses nicotinamide adenine dinucleotide (NAD+) to form PAR polymers, which are then transferred to acceptor proteins, including PARP1 itself [139,140,141]. In UWB1.289 cells, these proteins were shown to localize in the nucleus [137]. Using recombinant PARP and HMGB3, Ma et al.’s in vitro study showed that an increase in the amount of HMGB3 leads to an augmented enzymatic activity of PARP1 [137]. The authors also suggested that the interaction between these proteins leads to a change in the kinetics of PARP binding to DNA and, consequently, to chromatin.

High levels of HMGB3 expression are associated with lymph node metastases and lowered overall chance of patient survival [137]. HMGB3 promotes ovarian cancer resistance to PARP inhibitors through direct interaction with PARP1 [137]. Thus, HMGB3 promotes drug resistance by regulating DNA damage response pathways in ovarian cancer [119,137].

HMGB3 enhances the stemness, proliferation, and metastasis of ovarian cancer cells. In addition, Ma et al.’s work has shown that the MAPK/ERK signaling pathway is involved in the HMGB3-mediated malignant progression of ovarian cancer [142], and inhibition of MAPK/ERK signaling counteracts the effect of high HMGB3 expression in ovarian cancer. These results indicate that HMGB3 is a promising target for the development of therapeutic strategies against ovarian cancer.

### 4.6. SOX9

Recently, a new mechanism for regulating the development of prostate adenocarcinoma (PCa), which involves the deregulation of SOX9, HMGB3, and NANOG, was discovered. Xu et al. demonstrated a direct physical interaction between HMGB3 and SOX9 [143]. SOX9 protein is a single HMG-box family member [144] that regulates transcription by recognizing the conserved DNA motif (A/T)(A/T)CAA(A/T)G of target genes through its HMG-box. SOX9 and HMGB3 are associated with the *NANOG* gene promoter in the region of −573 to −568, while the binding of SOX9 is facilitated by HMGB3. The cooperation between the two proteins, mediated through the A domain of HMGB3 protein, facilitates the transcription of *NANOG*. The transcription factor NANOG is required for the establishment and maintenance of pluripotency and self-renewal of embryonic stem cells (ESCs) in concert with various factors, including OCT4, SOX2, and KLF4 [143]. Furthermore, NANOG is expressed in various human malignancies (including brain, colon, breast, liver, and prostate cancers) and promotes cell proliferation, migration, invasion, and therapy resistance [145,146,147,148]. Thus, HMGB3/SOX9-mediated NANOG induction may lead to increased viability of prostate adenocarcinoma cells [143]. Additionally, HMGB3 may function as a coactivator of SOX9, forming a new regulatory mechanism that activates NANOG transcription. Co-overexpression of SOX9 and HMGB3 is associated with prostate cancer progression and poor prognosis.

### 4.7. HMGA1

It was shown by 2-DE and LC-MS/MS that HMGA is a molecular partner of HMGB3. HMGB1, HMGB2, HMGB3, and HMGA1 are involved in RNA processing, whereby their action presumably affects chromatin structure and DNA-dependent processes [149]. A GEO-based prognostic model consisting of six gene products (ARHGAP11A, H1.4, HMGB3, LRIG1, PRR11, and COL4A1) has been shown to be a reliable predictor of esophageal cancer. The new model correlates with the prognosis of patient survival in GC esophageal cancer [150]. In the work of Wu et al. [151], the authors analyzed the expression of several HMG family members using qRT-PCR and various publicly available databases on esophageal cancer cell lines. HMGA1, HMGA2, HMGB1, HMGB2, HMGB3, HMGN1, HMGN2, and HMGN4 showed high expression levels in GC tissues compared to normal gastric tissues. At the same time, a positive correlation was found between various proteins of the HMG family: HMGA1 and HMGA2, HMGB1, HMGB2, HMGB3, HMGN1, HMGN2, and HMGN4; HMGA2 and HMGB1, HMGB3, HMGN1, and HMGN2; HMGB1 and HMGB2, HMGB3, HMGN1, HMGN2, and HMGN4; HMGB2 and HMGB3, HMGN1, HMGN2, HMGN3, and HMGN4; and HMGB3 and HMGN1, HMGN2, HMGN3, and HMGN4. The most prominent factors negatively associated with HMGB3 included C16orf89, ACKR1, and GFRA1, while the top factors positively associated with HMGB3 level were DKC1, CDK1, and CENPA. The expression level of HMGB3 had a strong positive correlation with CD274 (PD-L1), CTLA4, LMTK3, and SIGLEC15 levels. HMGB3 mRNA expression in gastric cancer correlated positively with immune infiltrates in Th2 cells and T helper cells and negatively with those in mast cells and B cells.

### 4.8. Receptors

In recent years, the involvement of HMGB proteins in innate and adaptive immune responses has attracted great interest. These proteins have been recognized as proinflammatory mediators during infection or sterile tissue injury, where their role is regulating innate immune responses [9,11,35,36,108]. HMGB1 and HMGB3 are currently considered universal sensors that can recognize damage-associated molecular patterns (DAMPs) and pathogen-associated molecular patterns (PAMPs) [76,152,153,154]. These new functions of the protein require interplay with a variety of cellular receptors. To date, a body of evidence has demonstrated HMGB proteins are directly or indirectly involved in the functioning of several receptors: receptors for advanced glycation end products (RAGE), Toll-like receptors (TLR), cluster of differentiation 24 (CD24), C-X-C chemokine receptor type 4 (CXCR4), N-methyl-D-aspartate receptor (NMDAR), T-cell immunoglobulin and mucin domain-3 (TIM-3), and triggering receptor expressed on myeloid cells 1 (TREM1) [26,76,155,156,157,158,159,160,161]. The functions of many of these receptors are associated with the redox status of HMGB1. There is less experimental evidence concerning the participation of HMGB3 in the functioning of cellular receptors (see Table 2). However, it has been reported that HMGB3 is involved in the functioning of TLRs, estrogen receptor (ER), progesterone receptor (PR), human epidermal growth factor receptor 2 (HER2), TREM1, and receptor-like kinases BAK1 and BKK1 [153,162]. In the following section, we have summarized experimental evidence of direct associations between HMGB3 and the TLR and TREM1 receptors.

#### 4.8.1. TLRs

In vertebrates, HMGB3 recognizes Toll-like receptors (TLRs) and activates NF-kB signaling and reactive oxygen species (ROS) production [163,164]. HMGB proteins can recognize and potentiate TLR2/4 to stimulate the release of cytokines and inflammatory factors, as clearly demonstrated for human HMGB3 [165].

The direct interaction between HMGB3 (like HMGB1 and HMGB2) and TLRs remains controversial. However, it has been reported that HMGB3 association with TLR3, TLR7, and TLR9 on the membrane leads to the production of ROS [163]. ROS induce and activate the NF-kB pathway to enhance the expression of vascular endothelial growth factor (vEGF) and interleukin-6 (IL-6). Additionally, ROS influence the activity of transforming growth factor β (TGFβ) and metalloproteinase 2/9 (MMP2/9), which ultimately promotes tumor growth and metastasis [163].

#### 4.8.2. TREM1

Inhibition of HMGB3 expression reduces cell viability, promotes apoptosis and cell cycle arrest, and suppresses migration and invasion of thyroid cancer cells. In papillary thyroid cancer (PTC), HMGB3 levels are elevated in both tumor and serum, which correlates with lymph node metastasis and the advanced tumor stage. Zhao et al. [155] observed a change in HMGB3 localization in the PTC due, at least in part, to hypoxia. Cytoplasmic HMGB3 activates nucleic acid-mediated TLR3/NF-κB signaling, and extracellular HMGB3 interacts with the transmembrane receptor triggering receptor expressed on myeloid cells (TREM1 and CD354) in thyroid cancer. Zhao et al. suggested a pro-oncogenic role of cytoplasmic and extracellular HMGB3 in thyroid cancer [155]. Thus, HMGB3 could be used as a potential circulating biomarker and therapeutic target in PTC and thyroid cancer.

### 4.9. Putative HMGB3 Partners

In addition to the described HMGB3′s partners, there are several proteins that have been predicted to physically interact with HMGB3 (Figure 8). Among those are PAX5, PAX6, MEOX1, MEOX2, SDCBP, TERF2iP, and CYP2B6, as well as another member of the HMGB group proteins—HMGB1.

*PAX* genes encode a family of transcription factors called “pair boxing” (PAX). A specific feature of these proteins is a highly conserved DNA-binding motif—the “paired box”. The PAX factors are important regulators during early development, and changes in their gene expression are thought to promote neoplastic transformation. For example, PAX5 is a B-cell lineage-specific activator protein expressed early in B-cell differentiation. PAX5 expression has also been found during the development of the central nervous system and testis, where it may play a role [166]. PAX6 is well studied and has been referred to in the literature as the master factor controlling the development of the eyes, sensory organs, and certain neural and epidermal tissues. In esophageal cancer cell lines, it was shown that PAX5 knockdown resulted in resistance to cisplatin, and the presence of methylation marks in the *PAX5* gene may be a diagnostic marker of cisplatin resistance, as well as of a poor survival prognosis [166]. In addition, PAX5 inhibits proliferation, promotes apoptosis, and activates p53 signaling in esophageal cancer [167]. Direct physical [128] interaction between PAX and HMGB3 has been predicted (Figure 8), but its functional significance has not been addressed.

Physical interaction with HMGB3 was also predicted for telomeric repeat-binding factor 2-interacting protein 1 (TERF2iP) [128]. The protein takes part in DNA replication, RNA-dependent DNA replication, DNA compaction, DNA metabolism processes, nucleosome assembly, and direct physical interaction with homeobox protein MOX-1 (MEOX), Syntenin-1 (SDCBP) protein, and heparin. Currently, neither direct experimental evidence confirming the interaction of the listed putative HMGB3 partners has been obtained nor have the biological roles of these interactions been established.

**Table 2:** Molecular partners of HMGB3.

Partners	Functions	References
DNA	HMGB1–3 proteins (DNA chaperones) bend DNA and facilitate its binding to transcription factors and various proteins.	[9,11,12,35,36,73,108]
	In addition, they recognize and preferentially bind to DNA regions with various structural abnormalities.	[9,11,56,118]
	Overexpression of HMGB3 can inhibit the DNA bending process and interfere with the binding of DNA and transcription factors.	[39]
RNA	HMGB3 binds RNA, facilitating their binding to pattern recognition receptors, such as TLRs, and control tat mRNA processing and translation. HMGB3 has been found in cytoplasmic ribonucleoprotein (RNP) complexes, which are formed in response to cellular stress.	[14,34,126,127]
p53	The interaction between p53 and DNA requires HMGB proteins.	[9,11,129,130]
	The impaired binding of HMGB1–3 to DNA interferes with the interaction between p53 and DNA, which leads to disruption of the antitumor activity of p53.	[39]
TLR3, TLR7, TLR9	In vertebrates, the interaction between HMGB3 and TLR3, TLR7, and TLR9, which occurs on the membrane, leads to upregulation of the production of ROS and activation of NF-kB signaling, which ultimately regulates tumor growth and metastasis.	[34,155,163,164]
	Activation of TLR2/4 by HMGB1–3 proteins stimulates the release of cytokines and inflammatory factors.	[165]
HOX	Increased expression of HOXB6, HOXB7, and HOXB8 in hematopoietic cell lines blocks myeloid differentiation. The co-expression of HMGB3 and HOXB6 also suggests a joint role of these proteins in erythropoiesis.	[131,132,133,134,135,168]
PARP1	Direct interaction between HMGB3 and the PARP1 protein, which plays a decisive role in DNA repair processes, was revealed. An increase in the amount of HMGB3 leads to increasing enzymatic activity on the part of PARP1.	[139,140,141]
	HMGB3 promotes ovarian cancer resistance to PARP inhibitors through direct interaction with PARP1.	[137]
SOX9	A direct physical interaction between HMGB3 protein and SOX9, which promotes the binding of SOX9 to NANOG, has been demonstrated. When HMGB3 binds to SOX9, NANOG transactivation is induced, which can lead to increased cell survival and migration of prostate adenocarcinoma cells.	[143]
PAX	PAX5 and PAX6 are tissue-specific transcription factors. PAX5 is associated with neuronal development, spermatogenesis, and B-cell differentiation. In esophageal cancer cell lines, PAX5 knockdown promotes resistance of tumor cells to cisplatin, inhibits proliferation, promotes apoptosis, and induces activation of p53 signaling. Functions of the PAX complex with HMGB3 are unknown.	[167,169,170]
HMGA1	As shown by 2-DE and LC-MS/MS, HMGA is a molecular partner of HMGB3. HMGB1, HMGB2, HMGB3, and HMGA1 are involved in RNA processing. Additionally, their action presumably affects chromatin structure and DNA-dependent processes.	[149,151]
HMGB1	Databases show a direct physical interaction between HMGB1 and HMGB3. This interaction has unknown functional significance.	[151]
TERF2	TRF2 is involved in the processes of DNA replication, RNA-dependent DNA replication, DNA packaging, DNA metabolic processes, and nucleosome assembly, among others. Databases show a direct physical interaction between TERF and HMGB3, although the functions are unknown.	[171]
NUP98	NUP98–HMGB3 acts as an oncogene responsible for rapid and transplantable MPD-like leukemia in recipient mice, which is associated with defects in myelomonocytic cell differentiation.	[172]
TREM1	Suppression of HMGB3 expression inhibits cell viability, promotes cell apoptosis and cell cycle arrest, and suppresses cell migration and invasion in thyroid cancer. Cytoplasmic HMGB3 activates nucleic acid-mediated TLR3/NF-κB signaling, and extracellular HMGB3 interacts with the transmembrane receptor TREM1 in PTC.	[155]
HIF1α	HMGB3 prevents mammosphere formation in breast cancer through binding to HIF1α.	[173,174,175]
	HMGB3 may promote ROS production and tumor cell proliferation by inducing HIF-1α expression. The silencing of HMGB3 attenuates HIF1α expression, leading to suppression of tumor cell proliferation.	[39]
SDCBP	The IntAct database shows a direct physical interaction between SDCBP and HMGB3 (Figure 8), although the functions are unknown.	[128]
MEOX	The databases show a direct physical interaction between MEOX and HMGB3 (Figure 8), with the functions unknown.	[128]
hTERT	The HMGB3/hTERT axis is important for resistance to radiation therapy in cervical cancer.	[123]
MAPK	HMGB3 promotes the development of malignant phenotypes and stemness of ovarian cancer through the MAPK/ERK signaling pathway.	[142]
Bcl-2/Bax	In gastric adenocarcinoma, downregulation of HMGB3 expression can dramatically suppress cancer cell proliferation, mainly through inducing G0/G1 blockade in tumor cells, regulating p53 and p21 signaling pathways, and reducing Bcl-2/Bax (anti-apoptotic factor/pro-apoptotic factor) levels.	[176]
MMP2, MMP9	Reducing the expression of HMGB3 leads to inhibition of invasion and migration of gastric cancer cells by suppressing the activation of MMP2 and MMP9.	[177]
ARHGAP11A, H1.4, LRIG1, PRR11 and COL4A1	A prognostic model consisting of six gene products (HMGB3, ARHGAP11A, H1.4, LRIG1, PRR11, and COL4A1) is a reliable predictor of esophageal cancer.	[150]
ER, PR	According to bioinformatics analysis, the co-expression of HMGB3 with ER and PR is associated with poor prognosis in breast cancer.	[107]
HMGN1, HMGN2, HMGN3, HMGN4	High rates of expression of HMGB3, HMGN1, HMGN2, HMGN3, and HMGN4 are shown in gastric cancer.	[151]
	Knockdown of HMGB3 suppresses the proliferation of cancer cells, stops their migration, and affects sensitivity to gastric cancer chemotherapy.	[39,177]
Heparin	The IntAct databases show a direct physical interaction between heparin and HMGB3 (Figure 8), with the functions unknown.	[128]

## 5. Expression of HMGB3

### 5.1. Regulation of HMGB3 Expression

A number of studies have established that the HMGB3 gene plays an important role in the development of malignant diseases [39,177,178,179]. In recent years, it has been shown that HMGB3 expression is regulated by various microRNAs and long non-coding RNAs [180,181], which act either by inhibiting the translation of the target RNA or inducing RNA degradation [39,182,183,184]. Dysregulation of microRNAs and long non-coding RNAs has been shown to be associated with the initiation and progression of various types of cancer [185,186,187,188,189,190,191]. In mammals, small RNAs are negative regulators of gene expression and, according to recent data, regulate the expression of more than 90% of proteins [183]. Some miRNAs are considered diagnosis biomarkers of tumors, including breast cancer [186,187,192]. In cancer cells, increased microRNA expression may promote cancer development by targeting tumor suppressor genes. In contrast, downregulation of antitumor microRNAs may lead to overexpression or activation of oncogenes.

Experimental data have shown that all members of the HMGB family are oncogenic, and suppression of their individual expression, using the RNA knockdown approach, clearly shows pronounced antitumor effects. The function of miRNAs is achieved through the regulation of downstream target genes: small RNAs bind to the 3′ non-coding region (3′UTR) of HMGB3 mRNA and regulate gene expression at the post-transcriptional level. Suppression of HMGB3 expression mediated by various small RNAs leads to a decrease in the aggressiveness of prostate cancer cells [193], causes cell death, and inhibits the migration and invasion of breast cancer cells [194,195], colon [196], stomach [177,197], pancreatic duct adenocarcinoma [198], and hepatocellular carcinoma [199]. Suppression of HMGB3 expression, mediated by various small RNAs, leads to a decrease in the aggressiveness of prostate cancer cells [181], causes cell death, and suppresses the migration and invasion of breast [182,183], colon [184], and gastric [149,185] cancer cells, as well as pancreatic ductal adenocarcinoma [186] and hepatocellular carcinoma [187]. Thus, in vivo and in vitro, inhibition of HMGB3 has been shown to impair cancer cell proliferation.

There are some examples of microRNA influence on HMGB3 expression. During the development and progression of cancer cells, HMGB3 expression is regulated by miR-206, miR-205, miR-27b, and miR-205-5p [121,193,194,200]. Studies of head and neck cancer have identified *HMGB3*, among other genes (*DDIT4*, *FOXD1*, *FXR1*, *FZD2*, *MINPP1*, *PAWR*, *PFN2*, and *RTN4R*), as a putative target of miR-30e-5p. The expression levels of these genes are associated with poor patient survival [201]. Reduced expression of miR-532-5p may contribute to a high rate of expression of HMGB3 in bladder cancer [202]. The underlying mechanism of miR-532-5p is related to its regulatory effects on HMGB3 expression and Wnt/β-catenin signaling [202].

According to the analysis of the TCGA database, another group of genes (*SPARC*, *MKI67*, *CENPF*, *CDK1*, *RHOU*, and *POLR2D*), which includes the *HMGB3* gene, is associated with a poor prognosis in cancer [193]. Compared with other genes in this group, the expression of HMGB3 in prostate cancer cells was most suppressed by the expression of miR-205-5p, which plays an important role in pathogenesis. In cultured bladder cancer cells, knockdown of circRNA TATA-box binding protein associated factor 15 (circTAF15) leads to suppression of cancer cell growth through downregulation of HMGB3 via direct suppression of miR-502-5p [178]. In vitro, by directly affecting the expression of mitogen-activated protein kinase 1 (MAPK1), miR-633 can inhibit proliferation, invasion, and migration of gastric cancer cells; arrest the cell cycle in the G1 phase; and induce apoptosis by regulating several genes, including HMGB3 [203].

In gastric adenocarcinoma cells, a reduction of HMGB3 expression can dramatically suppress the proliferation of cancer cells, mainly due to the induction of G0/G1 arrest in tumor cells, regulation of p53 and p21 signaling pathways, and the reduction of the level of anti-apoptotic factor Bcl-2/pro-apoptotic factor Bax [151,176]. Inhibition of HMGB3 expression leads to decreased viability, proliferation, migration, and invasion of cells in breast cancer [121], cervical cancer [204], esophageal cancer [205], non-small-cell lung cancer (NSCLC) [206,207,208], and stomach cancer [177]. These results suggest that HMGB3 is a novel tumor diagnostic and prognostic marker protein [39].

Recently, Sharma et al. studied the effect of miR-142-3p on the death of human cervical cancer [209] and breast cancer [210] cells. These works showed that HMGB3 is a direct target of miR-142-3p. Overexpression of HMGB3 and other HMG family members (HMGA1, HMGA2, and HMGB1) is directly associated with increased tumor size, metastasis, and poor response to radiation therapy in some types of cancer (including squamous cell carcinoma of the esophagus, colorectal cancer, and cervical cancer), as well as with a poor prognosis. Simultaneous knockdown of the HMGA1, HMGA2, HMGB1, and HMGB3 leads to a more significant effect than suppression of each of them separately. The authors showed that binding of miR-142-3p to the 3′ non-coding region (3′UTR) in HMGA1, HMGA2, HMGB1, and HMGB3 mRNAs leads to apoptosis of tumor cells, as well as to decreased proliferation, migration, and invasion. Thus, miR-142-3p directly targets HMGA1, HMGA2, HMGB1, and HMGB3, inhibiting them through reduction of cell viability, colony formation, migration, and invasion, as well as induction of apoptosis of tumor cells, particularly cervical cancer cells [209].

Several studies have shown that HMGB3 is a direct target of miR-27b [121] and miR-145-5p in breast, cervical, and esophageal cancer cells. Both miR-27b and miR-145-5p inhibit the expression of HMGB3 and reduce the viability, proliferation, migration, and invasion of tumor cells [121,204,205]. HMGB3 can accelerate the proliferation and colony formation of NSCLC cells, as well as reduce the apoptosis of these cells through the miR-145/HMGB3 signaling pathway, suggesting that HMGB3 can be considered a new marker of poor prognosis for NSCLC patients [208,211]. By targeting HMGB3, miR-27b can regulate the epithelial–mesenchymal transition (EMT) process and reverse resistance to several drugs, including the anticancer drug tamoxifen [121]. Histone deacetylase 3 increases the expression of HMGB3, promoting the immune escape of breast cancer cells by suppressing the microRNA-130a-3p axis [212]. In breast cancer, acting on HMGB3, miR-145-5p can inhibit the proliferation, invasion, and migration of cancer cells and also promote their apoptosis.

The poor prognosis for patients with NSCLC with high rates of HMGB3 expression may be due to increased glycolysis under hypoxic conditions, which is provided by the activity of miR-615-3p/HMGB3 signaling axis. Further, hypoxia stimulation may increase HMGB3 expression and promote malignant cell proliferation [39,211,213]. However, regulatory pathways can be different. In the cases of lung [214] and gastric cancer [215], HMGB3 is regulated by miR-513b through the mTOR signaling pathway [214]. miR-758 triggers tumor growth and metastasis in cervical carcinoma through interaction with HMGB3 and the Wnt/β-catenin signaling pathway [204]. In the case of bladder cancer, decreased expression of microRNA-532-5p activates the HMGB3/Wnt/β-catenin signaling pathway, leading to increased proliferation and invasion of tumor cells [202]. In nasopharyngeal carcinoma, HMGB3 may regulate tumor development through the LncRNA-SNHG5/miR-1179 signaling axis. Suppression of HMGB3 can effectively inhibit the proliferation and metastasis of these cells [216]. In lung adenocarcinoma, miR-5195-3p can complementarily bind to the 3′UTR of HMGB3 [217], whereby an increased level of miR-5195-3p expression leads to a decrease in HMGB3 expression. circRUNX1 promotes cancer cell progression by enhancing proliferation and metastasis through regulation of the miR-5195-3p/HMGB3 axis [217].

Several studies have shown that miR-30a-5p, which has been associated with a good survival prognosis of patients with certain malignancies [218], negatively regulates the expression of HMGB3 [219]. For example, in NSCLC, HMGB3 may act as an oncogene, and its increased expression can indicate poor outcomes for NSCLC patients [219], while suppression of HMGB3 expression by miR-30a-5p improves survival prognosis. Hsa-miR-30a-5p negatively regulates HMGB3 expression [219] and is associated with favorable patient survival prognoses [218]. Using A549 cell lines, it was shown that knockdown of HMGB3 in NSCLC leads to a decrease in the rate of colony formation and promotes apoptosis of tumor cells [208]. Suppression of HMGB3 expression inhibits proliferation and migration while promoting apoptosis in the placenta during fetal growth restriction [220].

In the work of Sun et al. on a rat sepsis model of acute lung injury caused by bacterial infection, the profiles of small nucleolar RNA host 16 (SNHG16), miR-128-3p, and HMGB3 were studied [221]. The authors showed that the extent of sepsis-induced lung injury is regulated by the miR-128-3p/HMGB3 axis. Downregulation of SNHG16 alleviates the disease, reduces inflammation, and helps reduce apoptosis by regulating the expression level of HMGB3. It has been shown that miR-195-5p has an anticancer effect not only in NSCLC [222] but in cervical cancer and squamous cell cancer of the oral cavity [223]. During the progression of NSCLC [181], a potential interaction between circ_0060937 and the miR-195-5p/HMGB3 axis was shown. Specifically, the knockdown of circ_0060937 prevented the development of NSCLC and glycolysis through the regulation of the miR-195-5p/HMGB3 signaling pathway. The MiR-195-5p/HMGB3 regulatory axis also plays an important role in glioma: miR-195-5p inhibits HMGB3 expression by directly targeting HMGB3 [181,224]. Potential targets were identified using bioinformatics analysis of the Starbase and Targetscan databases. MiR-195-5p binding sites were found in HMGB3, HMGA1, and HMGA2 [224]. The knockdown of miR-195-5p leads to increased cell proliferation, suppresses cell apoptosis, and activates Wnt/β-catenin signaling. Wnt-mediated suppression of HMGB3 also suppresses tumor cell proliferation [224]. MiR-424-5p binds to HMGB3 in acute injury of human pulmonary alveolar epithelial cells stimulated by lipopolysaccharides [225] and in patients with sepsis-induced lung damage [221,225]. In an in vitro model of acute injury in human pulmonary alveolar epithelial cells, depletion of circSLCO3A1 was shown to suppress apoptosis and inflammation by reducing proinflammatory cytokine production and inhibiting cellular apoptosis by synergizing with the miR-424-5p/HMGB3 axis [225].

HMGB3 may promote the progression of colorectal cancer (CRC) through the activation of WNT/β-catenin/c-Myc signaling [226]. Recent work by Gong et al. showed that the expression level of HMGB3 correlates with the proliferation and migration of tumor cells in CRC, and HMGB3 knockdown can reduce both the proliferation and migration of these cells [81]. Recently, it was shown that exosomes with increased levels of miR-200b-3p—derived from hypoxic cancer-associated fibroblasts from the tumor environment, which inhibit the HMGB3/b-catenin/c-Myc axis—can sensitize CRC cells to the chemotherapy drug 5-fluorouracil (thymidylate synthase inhibitor) [227]. The oncogenic role of long non-coding RNA (200 nt) LINC00857 is mediated by the miR-150-5p/HMGB3 axis. In CRC tumor samples, the level of HMGB3 positively correlated with the expression level of LINC00857 but negatively correlated with the expression level of miR-150-5p. LINC00857 promotes HMGB3 expression by competitively binding to miR-150-5p. The LINC00857/miR-150-5p/HMGB3 regulatory axis plays a fundamental role in regulating the malignant phenotype and tumorigenesis of CRC [180].

The work of Ji et al. demonstrated that HMGB3 binds miR-216a-3p [228], which is a tumor suppressor in colon [196], gastric [197,229], and breast cancers [195], as well as in pancreatic ductal adenocarcinoma [198]. Additionally, high levels of HMGB3 expression promote the development of colon cancer. In contrast, by targeting HMGB3, miR-93 and miR-429 can suppress the development of CRC [230,231]. The circ-IGF1R/miR-362-5p/HMGB3 regulatory axis is also involved in the progressive development of CRC [232]. Gao S et al. showed in vivo that circ-IGF1R promotes tumor growth and glycolytic metabolism by activating the Wnt/β-catenin pathway through the regulation of the miR-362-5p/HMGB3 axis. Suppression of circ-IGF1R blocks the Wnt/β-catenin pathway by inhibiting miR-362-5p-mediated HMGB3 expression, which accelerates CRC progression. In NSCLC, circ-IGF1R suppresses cancer cell invasion and migration [233].

Another mechanism for influencing the expression of HMGB3 was reported for miR-200b. In gastric cancer, miR-200b overexpression stimulates proliferation, cell invasion, and EMT, thereby suppressing tumor development and increasing sensitivity to the anticancer drug cisplatin through negative regulation of HMGB3 [234]. Thus, miR-200b-3p (as well as miR-200c-3p) plays a negative regulatory role in relation to HMGB3 and can significantly inhibit tumor development [39,235].

In prostate cancer tissues and cells, the expression of long non-coding RNAs Sox2-OT (SOX2 Overlapping Transcript) and HMGB3 increases, while the expression of miR-452-5p decreases. Knockout of the Sox2 leads to suppression of the activity of the miR-452-5p/HMGB3 signaling axis and can cause inactivation of the Wnt/β-catenin signaling pathway, which can effectively slow the growth and metastasis of prostate cancer cells [236].

HMGB3 is expressed at high rates in stem cells and cancer cells and is rarely upregulated in normal adult tissues, making it a promising therapeutic target [2,237]. HMGB3 regulates a correct balance between self-renewal and differentiation of hematopoietic stem cells (HSCs). HMGB3 deficiency results in increased self-renewal capacity of HSCs, which can lead to leukemia [135,168], with which HMGB3 is closely associated in terms of occurrence and development. In addition, HMGB3 may cause relapse and chemotherapy resistance in acute lymphoblastic leukemia [172,238]. In a mouse model of human acute myeloid leukemia, it was found that leukemia stem cells (LSCs) are closely associated with the expression of mixed lineage leukemia (MLL) oncogenes and are capable of self-renewal via a mechanism similar to that in embryonic stem cells (ESCs). HMGB3 is involved in the regulation of LSCs through the same mechanisms. Maintenance of LSCs and increased expression of ESC-specific genes promotes the proliferation of human tumor cells and their self-renewing precursors, such as cancer stem cells, forming recurrent tumors with a high rate of metastasis and contributing to the emergence of resistance to anticancer drugs [199,239,240].

### 5.2. Expression of HMGB3 in Tissues and Organs

Although HMGB1–3 are known as chromatin-binding proteins, they are found in both the nucleus and the cytoplasm [2,215]. Experimental data have demonstrated that HMGB1, HMGB2, and HMGB3 are ubiquitously expressed in mammals during embryonic development but differ in expression patterns in adults [3,241,242]. Table 3 summarizes the RNA expression levels of human HMGB1, HMGB2, and HMGB3. In some tissues, high levels of expression of these proteins are associated with very low levels of RNA expression (Table 3). In the case of HMGB3 protein, a high level of RNA expression is accompanied by medium (e.g., placenta) or low (e.g., ovary) levels of the protein expression, and vice versa, reduced HMGB3 RNA expression is associated with increased expression of HMGB3 protein in the respiratory system.

**Figure 9:**
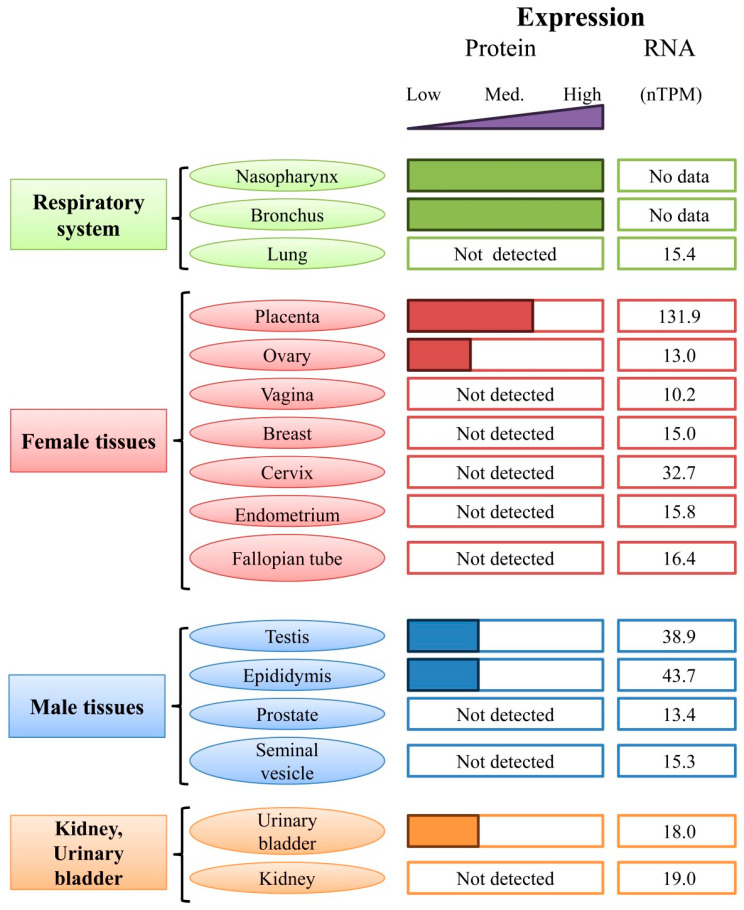
**HMGB3 RNA and protein expression.** Expression levels of HMGB3 protein (green, blue, red, and orange rectangles) and of RNA (transparent rectangle) in the human tissues and organs. The highest level of protein expression is observed in the respiratory system (green), and the lowest is observed in the bladder (orange). The expression differs between men (blue) and women (red): in women, an average level of HMGB3 expression has been shown in the placenta, while in men, the protein is expressed at a low level in testis and epididymis. nTPM means number of transcripts per million. The figure is based on data from the Human Protein Atlas [243].

Table 3 shows data on the expression levels of HMGB1-3 in various human organs and tissues. High levels of HMGB1 expression are observed in almost all human tissues and organs. Average levels of HMGB1 expression have been detected in the tissues of the endocrine system (parathyroid and adrenal glands), while low expression has been reported in the seminal vesicle. The gastrointestinal tract, male tissues, skin, and all bone marrow and lymphoid tissue organs are characterized by high levels of HMGB2 expression. Endocrine tissues, the respiratory system, the proximal digestive tract, the kidney, the bladder, female tissues, and muscle tissues show moderate levels of HMGB2 expression. The brain, liver, gallbladder, and pancreas, as well as connective and soft tissues, are characterized by low levels of HMGB2 expression. Figure 9 shows RNA and protein expression data for HMGB3 in various human tissues and organs. HMGB3 protein is highly expressed in the respiratory system (nasopharynx and bronchi) but not expressed in the lungs. The lowest levels of HMGB3 expression are observed in the kidneys, the bladder, and male tissues. The placenta shows moderate levels of this protein, while the protein is expressed at low levels in males. According to some data, HMGB3 expression in adult organisms is limited to hematopoietic stem cells of the bone marrow [135,168]. Other experiments have demonstrated a high level of human HMGB3 expression not only in the placenta but also in the bone marrow, colon, endometrium, brain, and liver, while the protein is practically absent in the pancreas and salivary glands [135,163,244].

The human *HMGB3* gene is located on the X chromosome (Xq28) [3,37] and is part of cluster 58, which includes 142 genes. HMGB3 mRNA has been shown to be present at relatively high levels in the erythroid and primitive bone marrow cells of adult mice. HMGB1 and 2 are expressed in all cell types, but HMGB3 is the only HMGB family member highly expressed in certain populations of hematopoietic cells [135,168], namely in lymphoid and myeloid progenitors, where the protein is associated with myeloid and B-cell differentiation. In addition, HMGB3 has been shown to regulate a balance between the processes of self-renewal and differentiation of hematopoietic stem cells [168,245].

In somatic cells, HMGB3 is expressed at a very low level; in stem and cancer cells, it is significantly upregulated. This feature allows for the consideration of HMGB3 as a prognostic marker and potential therapeutic target for various oncological diseases [39,237]. It is important to note that HMGB3 is an oncogene that promotes, through various mechanisms, initiation, development, and resistance to chemotherapy in breast cancer [210], colorectal cancer [81], thyroid cancer [91], neuroblastoma [246], nasopharyngeal carcinoma [247], and cervical cancer [179].

In mice, all proteins of the HMGB group are expressed in early mouse embryos, while HMGB1 continues to be expressed in adults [248]. HMGB2 and HMGB3 seem to be capable of functionally replacing HMGB1 during embryogenesis, as HMGB1 knockout embryos reach pregnancy to term [241]. However, as these mice show severe abnormalities (small size, elongated hind legs, ruffled and disorganized fur, and lack of fat) and die within hours after birth due to ineffective activation of glucocorticoid receptor-responsive genes, HMGB2 and HMGB3 do not seem to fully substitute HMGB1 function after birth [3,135]. HMGB1 knockout mice die within hours after birth due to ineffective activation of glucocorticoid receptor-responsive genes and severe organ abnormalities [241]. HMGB2 is present in adult mice in the thymus, testis, and lymphoid tissues, and the protein is also expressed in all immortalized human and mouse cells [242,248]. HMGB3 is expressed in primitive hematopoietic cells of the bone marrow [135,168].

## 6. Conclusive Remarks

Proteins with high electrophoretic mobility are the second most abundant group of nuclear proteins after histones. Along with histones, HMG proteins are necessary for proper chromatin folding as they interact with nucleosomes, internucleosomal DNA, linker histones of the H1 family, and other proteins. Members of a sub-family of HMG proteins called HMGB, including HMGB3, take an active part in almost all functionally significant cellular processes associated with DNA: transcription, repair, recombination, and replication. In addition, these proteins act as cytokines during injury and in response to inflammatory and infectious processes.

Another interesting aspect of studying HMGB3 regards its structure. All these proteins have a short N-terminal region, two DNA-binding domains, and a long, negatively charged C-terminal region. The C-terminal region modulates the interaction between these proteins and nucleic acids, as well as other protein molecules. According to their a.a. sequence, the proteins are characterized by a high degree of homology. However, the length of their C-terminal region is different. HMGB3 protein has the shortest C-terminal sequence, and this undoubtedly affects its functions and, primarily, the interaction with its partners. A shortened C-terminus should result in increased affinity with nucleic acids. Using the recombinant HMGB1 protein, consisting of only two DNA-binding domains, it was shown that when interacting with DNA, both domains take part in DNA binding, whereas the intact molecule interacts with DNA through only one domain [11,12,110,249,250,251]. However, comprehensive studies on the structure of HMGB3 protein itself and its complexes with DNA have yet to be conducted. HMGB3 (like HMGB1 and HMGB2) is not only a nuclear protein actively involved in all DNA-dependent processes. It has been shown that, in the cell nucleus, HMGB3 (like other members of the group) behaves as a DNA chaperone. Outside the cell, HMGB3 interacts with various receptors on the cell surface, for example, TLR3, TLR7, and TLR9 [155,163], thereby participating in the transmission of the immune response and acting as a signaling molecule during inflammation [35]. In addition, analysis of HMGB1 and HMGB2 [9,73] showed that, despite the high degree of homology in the a.a. sequence of the two, these proteins differ in both the position of PTMs and the secondary structure, which also affects the interaction between HMGB1 and HMGB2 and DNA and other proteins. More thorough studies of the structure and functions of HMGB3 will help address these and other areas that are yet unknown, such as why cells need proteins that are so similar in structure, sub-cellular/intercellular localization, and functions.

A direct connection was found between the expression of HMGB3 and the course of various oncological processes. Furthermore, the level of expression is significantly lower in normal tissues than in malignant tissues. HMGB3 predominantly accumulates in the nucleus of tumor cells and partially passes into the cytoplasm. The level of HMGB3 expression is considered a prognostic marker of the survival of patients with cancer, including esophageal [150,252], breast [107], NSCLC [211], and prostate cancers [143]. A positive association has been shown between HMGB3 expression and the identity of B cells, dendritic cells, and neutrophils [107].

A number of studies have examined the role of HMGB3 in the diagnosis, development, and treatment of oncological diseases [39,253,254]. Although HMGB1 is known to be involved in cardiovascular diseases, the possible role of HMGB3 is yet to be studied.

Suppression of HMGB3 expression appears to be a promising approach to address resistance to certain anticancer drugs (e.g., tamoxifen, cisplatin, and 5-fluorouracil), which may create the opportunity for more successful treatments of a number of oncological diseases. All three proteins of the HMGB group can be considered potential targets for the therapy of various diseases; therefore, the development of HMGB3 inhibitors is becoming a highly relevant pursuit.

## Figures and Tables

**Table 1:** High-mobility group (HMG) superfamily.

Protein Name		Description
Old Nomenclature (before 2001)	Current Nomenclature (after 2001)	
HMG-1	HMGB1	Proteins contain structural and functional motifs, known as HMG-box. HMG-Box binds DNA in the minor groove, inducing DNA that bends toward the major groove. The proteins containing HMG-box preferentially bind DNA regions with various structural abnormalities.
HMG-2	HMGB2	
HMG-2a (chicken)	HMGB3	
ssHMG-2b (chicken)	HMGB2	
HMG-3	-	
HMG-4	HMGB3	
(Unknown until 2008)	HMGB4	
HMG-14	HMGN1	The proteins contain structural and functional motifs, known as nucleosomal-binding domain (NBD). Proteins specifically recognize the overall structure of the nucleosome.
HMG-17	HMGN2	
HMG-I	HMGA1a	The functional motif of the HMGA family is called the “AT-hook”. ATH domains bind AT-rich DNA regions with varying affinities but very little DNA sequence specificity.
HMG-Y	HMGA1b	

**Table 3:** Levels of RNA/protein expression in HMGB1 (ENSG00000189403), HMGB2 (ENSG00000164104), and HMGB3 (ENSG00000029993) in an adult organism vary. RNA expression for all three proteins was detected in all tissues. The table is based on data from the Human Protein Atlas [243].

Tissue	RNA/Protein Expression		
	HMGB3	HMGB2	HMGB1
Female tissues	high/medium	low/medium	medium/high
Endocrine tissues	high/–	very low/medium	medium/high
Liver and gallbladder	high/–	very low/low	medium/high
Male tissues	medium/low	low/high	medium/high
Gastrointestinal tract	medium/–	low/high	medium/high
Muscle tissues	medium/–	very low/medium	medium/high
Bone marrow and lymphoid tissues	medium/–	high/high	high/high
Respiratory system	low/high	very low/medium	medium/high
Kidney and urinary bladder	low/low	very low/medium	medium/high
Brain	low/–	very low/low	medium/high
Proximal digestive tract	low/–	very low/medium	medium/high
Skin	low/–	very low/high	low/high
Pancreas	very low/–	very low/low	low/high
Connective and soft tissue	very low/–	very low/low	medium/high
Eye	very low/–	low/–	low/–

## Data Availability

The data presented in this study are available in the article.
